# Exploring Regional Fine Particulate Matter (PM_2.5_) Exposure Reduction Pathways Using an Optimal Power Flow Model: The Case of the Illinois Power Grid

**DOI:** 10.1021/acs.est.2c08698

**Published:** 2023-05-16

**Authors:** Ahmad Bin Thaneya, Arpad Horvath

**Affiliations:** Department of Civil and Environmental Engineering, University of California, Berkeley, California 94720, United States

**Keywords:** electricity, power flow, air quality, human health, exposure

## Abstract

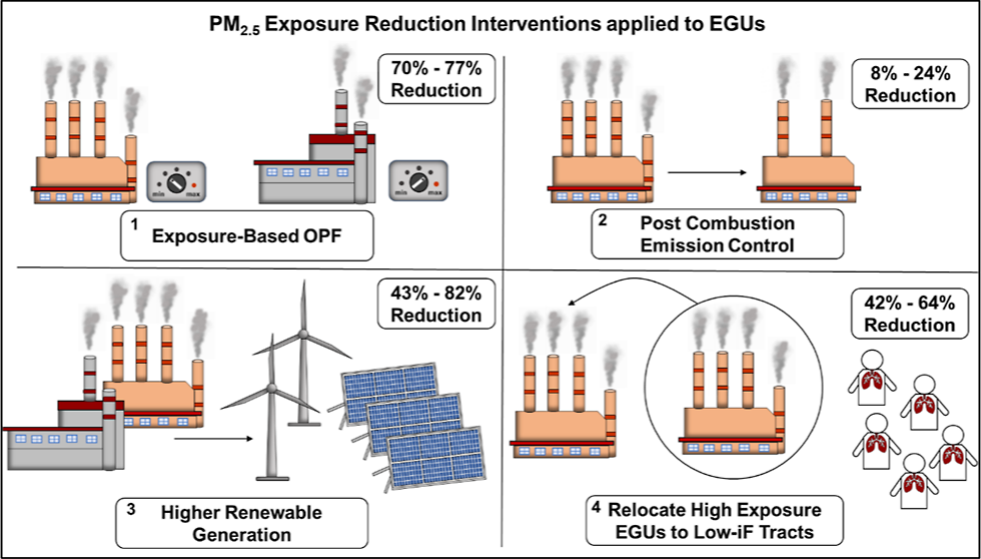

This work develops an exposure-based optimal power flow model (OPF) that accounts for fine particulate matter (PM_2.5_) exposure from electricity generation unit (EGU) emissions. Advancing health-based dispatch models to an OPF with transmission constraints and reactive power flow is an essential development given its utility for short- and long-term planning by system operators. The model enables the assessment of the exposure mitigation potential and the feasibility of intervention strategies while still prioritizing system costs and network stability. A representation of the Illinois power grid is developed to demonstrate how the model can inform decision making. Three scenarios minimizing dispatch costs and/or exposure damages are simulated. Other interventions assessed include adopting best-available EGU emission control technologies, having higher renewable generation, and relocating high-polluting EGUs. Neglecting transmission constraints fails to account for 4% of exposure damages ($60 M/y) and dispatch costs ($240 M/y). Accounting for exposure in the OPF reduces damages by 70%, a reduction on the order of that achieved by high renewable integration. About 80% of all exposure is attributed to EGUs fulfilling only 25% of electricity demand. Siting these EGUs in low-exposure zones avoids 43% of all exposure. Operation and cost advantages inherent to each strategy beyond exposure reduction suggest their collective adoption for maximum benefits.

## Abstract 2

Human exposure to fine particulate matter from electricity generation emissions remains a global health problem. This study develops an electricity dispatch model to explore different ways system operators can mitigate exposure while still balancing operational costs and network stability.

## Introduction

Long-term exposure to fine particulate matter (PM_2.5_) is a major environmental health risk, leading to an increased incidence of premature mortality due to cardiovascular disease, stroke, respiratory diseases, and lung cancer.^1−6^ The 2019 Global Burden of Disease study attributes 7.3% (4.1 million) of total global deaths to PM_2.5_ exposure, making it the leading environmental contributor to the global burden of disease.^7^

Estimates show that energy consumption accounts for 57% of all health impacts associated with PM_2.5_ human exposure in the United States.^8^ However, health damages from the electricity generation sector have been declining. Between 2008 and 2014, the gross external damages of U.S. utilities fell by almost 50% due to environmental regulations and market prices driving a major transition away from coal and toward natural gas (NG) and renewables.^9^ Despite these reductions, electricity generation units (EGUs), specifically coal-fired power plants, are estimated to remain the largest contributor of PM_2.5_-related health damages from elevated (i.e., high-smokestack) sources, where sulfur oxide (SO_*x*_) emissions cause 58% of damages (11% of all-sector damages).^8^ PM_2.5_ exposure due to electricity generation still causes approximately 10,000 premature deaths annually in the United States.^10^ PM_2.5_ exposure due to electricity generation remains an even larger health hazard globally. Ambient PM_2.5_ pollution is the 4th largest contributor to deaths in China and the 5th in India, where the electricity generation sector contributes to 520,000 annual premature deaths (39% of all PM_2.5_-related deaths) and 270,000 (32% of all PM_2.5_-related deaths), respectively.^11^ In Europe, coal-fired power plants lead to 33,900 PM_2.5_-related premature deaths annually.^12^ This necessitates the need for continued intervention within the electricity generation sector through better management of dispatchable EGUs and the continued integration of renewables. This research aims to contribute to that goal by building a comprehensive electricity generation dispatch model with an integrated health damage cost component that can be used for short- and long-term planning by system operators.

The health, economic, and equity implications of PM_2.5_ exposure due to electricity generation in the United States have been assessed in previous work.^13−19^ The effects of intervention strategies for reduced PM_2.5_ exposure such as fuel-switching,^14^ optimal plant siting,^20^ and higher renewable integration^13^ have also been studied at a national scale. Analyzing these effects has led to the development of variations of economic dispatch (ED) models with integrated health and climate components. The goal of ED models is to minimize system costs while also accounting for constraints related to power balance and EGU limits (i.e., generation capacity).^21^ A comprehensive but not exhaustive list of these works is described by Deetjen and Azevedo.^14^ Studies include Kerl et al.,^22^ which integrated linearized emission-concentration sensitivities into a dispatch model to minimize system operating costs and PM_2.5_-related health damages from SO_*x*_ emissions in Georgia. The formulation in Kerl et al.^22^ also accounted for unit-commitment and generator-ramping constraints. Deetjen and Azevedo^14^ used a reduced-order dispatch model to assess the climate, health, and economic impacts of replacing coal-based electricity with existing, underutilized NG capacity. Sergi et al.^20^ developed an optimization framework that considers climate and health implications when siting locations for replacing power plants with new wind, solar, or NG plants. The consensus from these studies points to a similar conclusion: incorporating health considerations into dispatch and planning models will lead to significant exposure reductions.

We build upon the existing literature by developing an alternating current (AC) optimal power flow (OPF) model^23^ that accounts for human health-related damages from primary and secondary PM_2.5_ exposure. An OPF formulation is an extension of the ED problem that includes network constraints such as transmission line loading limits, transmission stability limits, bus voltage limits, and generator reactive power.^21^ Expanding the existing reduced-complexity health-based dispatch models to an OPF formulation is an essential development given that OPF models are instrumental tools used by Independent System Operators (ISO) for long-term capacity planning and minute-by-minute adjustment of real and reactive power dispatch.^24^ OPF models and their various formulations are applied to operating objectives such as safeguarding the reliability, security, and stability of electricity generation and transmission. The implementation of intervention strategies to complex infrastructure systems, such as power systems, requires understanding the potential effects these strategies may have on primary operating system parameters. The mitigation potential of these interventions must be rooted in realistic system constraints to ensure their feasibility for adoption. The modeling framework developed in this study provides system operators with the ability to incorporate human health considerations into short- and long-term planning while still prioritizing other power system cost and stability parameters. The results of this modeling exercise can also aid policymakers in mandating feasible systemwide operations and design changes for safeguarding human health.

The main contributions of this work lie within leveraging the strengths of OPF models to develop a decision-making framework that exists at multiple scales: (1) modeling detailed day-to-day electric power system operations and their associated health impacts from five different PM_2.5_ pathways, and (2) exploring long-term system planning to reduce PM_2.5_-related health impacts from electricity generation. The model’s decision-informing power is demonstrated through a case study of a representative Illinois-based electric power grid network. To our knowledge, no previous electricity dispatch model has accounted for OPF constraints while minimizing PM_2.5_ exposure at a regional scale. The importance of accounting for transmission constraints is demonstrated by quantifying the additional operational costs and health damages that arise from transmission losses. This is done by comparing the results of the AC OPF to the linearized direct current (DC) OPF approximation of the same network that ignores transmission constraints and reactive power flow. Once baseline system operating costs and exposure damages are established, potential pathways to reduce regional PM_2.5_ exposure damages are explored, including (1) incorporating PM_2.5_ exposure damages into the OPF objective function, (2) retrofitting EGUs with the best-available (i.e., highest efficiency) post-combustion NO_*x*_ and SO_*x*_ pollutant control technologies, (3) higher renewable generation, and (4) relocating and fuel-switching of high-polluting EGUs.

## Materials and Methods

An exposure-based OPF requires several modeling components: (1) an optimization dispatch model used to obtain an electric power dispatch schedule and other operating/emission characteristics, (2) a source-receptor (SR) matrix for primary and secondary PM_2.5_ formation, (3) geospatial analysis for generating exposure profiles, and (4) a health damage model.

Exposure impacts due to EGU emissions are estimated using results from three OPF optimization scenarios: (1) a baseline scenario considering operational dispatch costs only, (2) an OPF considering exposure damages only, and (3) an OPF considering dispatch costs and exposure damages collectively (hereafter: the all-cost scenario). The baseline scenario is assumed to closely approximate actual system conditions where an operator seeks to minimize dispatch costs. The second scenario dispatches EGUs to minimize PM_2.5_ exposure damages. The third optimization scenario assumes that the dispatch cost model internalizes the exposure externality of electricity generation, which allows system operators to minimize the system’s induced exposure damages in addition to general operational costs. Other intervention strategies will be analyzed through modified runs of all three optimization scenarios.

A network representing the Illinois power grid has been constructed from several data sources and is used to demonstrate the model’s utility. Illinois was chosen because its network of EGUs contributes to large exposure damages within and across its borders; it is estimated that only Indiana, Texas, and Pennsylvania contribute to more EGU-related deaths.^16^ However, Illinois also has a diversified electricity mix, including coal, nuclear, NG, oil, and renewables, which signifies its feasibility and potential for reduction.^25^ Each model run simulates 12 averaged 24 h periods for each month of 2019. The results of each one-day simulation are assumed to apply to every day of that month, thus resulting in 8760 h of simulation of the year. This resolution captures monthly changes in loads and operational costs and allows us to infer general trends without incurring large computational times. Developing an electric power system network for an OPF requires several spatial and parametric data inputs. These include network bus, generator, substation and transformer, transmission line, and load demand data. Although the power network is limited to Illinois, the exposure domain for the optimization includes its 10 nearby states to capture the impacts of secondary PM_2.5_ exposure. Figure S1 in the Supporting Information displays a map of the entire exposure domain.

### OPF Formulation

OPF models and formulations are extensively documented in the literature.^26,27^ The mathematical formulation and notation used are presented in the Supporting Information and adopted from Frank and Rebennack.^24^Table S1 in the Supporting Information summarizes the model’s variables and parameters.

An OPF is formulated as an optimization problem by combining fundamental AC power flow equations with an objective function. The power flow equations are used as OPF constraints to ensure that the optimal solution is a feasible one. Objective functions of OPF problems typically minimize total costs of electricity generation by controlling real power injections at generator buses (i.e., the optimization variables), while the constraints ensure that the system is operating within safety limits. Constraints include setting limits on bus voltage magnitudes, voltage angles, and maximum current loading on network branches.

Results are compared against a DC OPF approximation to quantify the additional costs and damages that arise from including network transmission constraints and reactive power flow. DC OPFs use a linearized version of the AC power flow equations, making the problem less computationally expensive to solve. The objective function of the DC OPF remains unchanged from that of the AC OPF one. The power system solver PyPower, a Python portal to MATPOWER, is used for solving the multiple OPF runs.^28^

### Network Setup and Data Description

**Figure 1:**
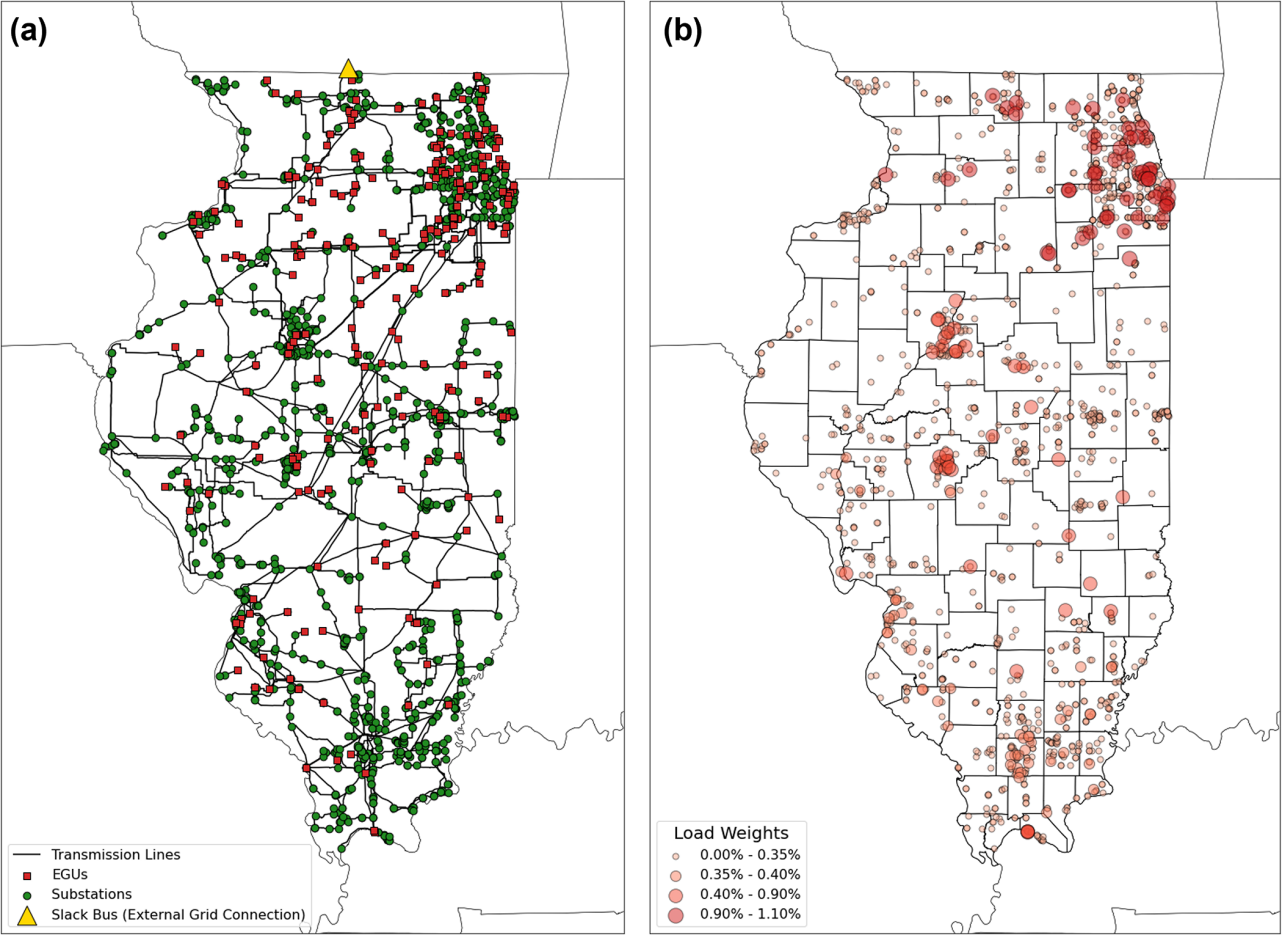
(a) Representation of the Illinois synthetic electric power grid containing network buses, transmission lines, EGUs, and the network slack bus. Transmission lines conduct electric currents between different network nodes. The nodes in the network represent substations which house transmission equipment that switch, transform, and regulate electric power. (b) Distribution of load weights among network substations. Given that the exact distribution of network load is not publicly available, several steps are taken to obtain a representative, spatially and temporally resolved load profile for this network. Power load demand is assumed to be consumed at the substation level, specifically at substations where voltage levels are stepped down to distribution-level voltages. Substations which meet that voltage criteria are identified and designated as load buses. Load weights are assigned based on the location of the load buses with respect to Illinois’ Electric Retail Service Territories. Higher load weights are observed for large Illinois cities such as Chicago in the northeast and Peoria and Springfield in the central part of the state.

A representation of the Illinois power system network was developed from several data sources following established methodology for constructing synthetic networks.^29−32^Figure 1a displays the network along with its several components, including network buses (with transformers),^33^ transmission lines,^34^ EGUs, and the network slack bus.^25^Figure 1b shows the distribution of load weights to the network substations, which were developed using data from Illinois’ Electric Retail Service Territories.^35^ Details regarding the development of the network and load profile can be found in the Supporting Information.^25,36,37^ Using a constructed network introduces error and uncertainty in the modeling framework and is not a definitive representation of the real-life network. Rather, it is an approximation used to infer trends and inform decision-making. The motivation behind this exercise is to demonstrate the model’s usefulness for system operators who hold the actual data. Model performance metrics are quantified to test the reliability of the model and its associated parameters in predicting observed EGU generation despite the approximations used.

### Exposure Damage Modeling

Incorporating health damages into the OPF requires an exposure damage cost function. We have previously developed an exposure-based traffic assignment model that minimizes PM_2.5_ exposure from modeled on-road vehicle emissions on a regional network.^38^ Herein, we employ a modified version of the exposure analysis framework developed in Bin Thaneya et al.^38^ A PM_2.5_ exposure damage function normalized per MWh of electricity generated for each EGU is developed through emissions,^25,39−42^ concentration,^8,43^ and intake modeling. Pollutant intake is an exposure metric that represents the mass of pollutants inhaled over time by the exposed population.^44−46^ Another useful metric is the intake fraction (*iF*) of an emission source, which is an efficiency metric that quantifies the total intake that would take place per unit of emissions.^47^ The exposure-based OPF model accounts for both primary and secondary PM_2.5_. Secondary PM_2.5_ emission precursors considered are NO_*x*_, SO_*x*_, volatile organic compounds (VOCs), and ammonia (NH_3_). Information regarding emission data is provided in the Supporting Information.

Annual average increases in ambient PM_2.5_ concentrations are quantified through the Intervention Model for Air Pollution (InMAP)^43^ Source-Receptor Matrix (ISRM),^8^ which holds linearized emission-concentration relationships developed from InMAP, a reduced-complexity air quality model. The ISRM is adopted since concurrently running an OPF and a chemical transport model (CTM) is computationally expensive. Another benefit of the ISRM is its variable grid resolution, which becomes progressively finer in areas with higher population densities but remains coarse in lower population density areas. This is ideal for exposure assessment since it allows for the modeling of finer pollutant concentration gradients in areas with large population numbers, while being computationally efficient in capturing long-range transport and secondary formation of PM_2.5._ ISRM data of each of the five PM_2.5_ species are augmented with population^48^ and breathing rate data^49^ before being spatially joined to the EGU network, resulting in an *iF* matrix denoted by ) for each pollutant (***u*** ∈ ***U***). Matrix rows (***M***) and columns (**Γ**) represent the set of study area census tract and network EGUs, respectively. Details of the approach used to develop the *iF* matrices are similar to those outlined in the Supporting Information of Bin Thaneya et al.^38^

Pollutant intake is translated into a monetized exposure damage cost function for each EGU normalized per MWh of electricity generated. Nonlinear concentration–response functions^1,50^ are computationally difficult to integrate into an OPF model. Instead, linearized PM_2.5_ intake effects and severity factor parameters^51^ are used to approximate the exposure damages. The parameter (**ν**) [$ per kgPM_2.5_ inhaled] represents an exposure factor that transforms PM_2.5_ intake into premature deaths and then monetizes the damages using the EPA-recommended value of statistical life (VSL).^52^ Once the OPF is run with the linearized parameters, the nonlinear exposure damage functions^1,50^ are used to generate final results.

The exposure damage cost function is shown in (1) and is denoted by (, where ***P***_***i***_^***G***^, the real power generation from the ***i*th** generator, represents the optimization variable.  is the second term in the complete OPF objective function shown in (2). The first term  is the operational cost obtained from the product of the per unit dispatch costs and the real power output of generator ***i***. **λ**_***ium***_ [kgPM_2.5_ inhaled per hour per g/h of pollutant emitted] is an element from the group of *iF* matrices (**Λ**) for pollutant ***u***, representing the amount of intake induced in exposure zone ***m*** due to a unit of emissions of pollutant ***u*** from generator ***i****.* To obtain the total intake from that generator, **λ**_***ium***_ is multiplied by the total emissions of pollutant ***u***, which is the product of the EGU’s emission factor (***E***_***iu***_) [g/MWh] and the hourly power generation (***P***_***i***_^***G***^) [MWh] from unit ***i***. Daily intake is transformed into a unit cost by multiplying the term with the exposure damage factor (**ν** ≈ $80 M per kgPM_2.5_ inhaled). Details regarding the development of this factor can be found in the Supporting Information. Running the OPF with the first term alone represents the baseline scenario. Running the OPF with only the second term represents the aggressive exposure mitigation scenario. Running the OPF with both terms is the all-cost scenario that internalizes the exposure externality of electricity generation within the cost model.
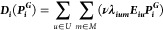
1
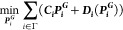
2

### Analysis of Exposure Reduction Strategies

The study explores four PM_2.5_ exposure mitigation strategies. Exposure reductions are compared against the baseline OPF run. The first strategy involves the two OPF runs (described in the Materials and Methods section) where the exposure damage costs are integrated into the OPF objective function. Three variations of each of the remaining strategies are developed: (1) post-combustion emission control^53,54^ including (i) adopting best-available SO_*x*_ control technology in coal-powered EGUs, (ii) adopting best-available NO_*x*_ control technology in all fossil fuel-powered EGUs, and (iii) adopting best-available pollutant control technologies for both pollutants in all fossil fuel-powered EGUs; (2) higher renewable generation^20,25,55−59^ including (i) a 2026 scenario (25% of total electricity generation), (ii) a 2040 scenario (50%), and (iii) a 2050 scenario (75%); (3) high-polluting EGU relocation^59,60^ where (i) EGUs are relocated and replaced as is, (ii) EGUs are relocated and replaced with a low-emission version of the same plant type, and (iii) EGUs are relocated and replaced with a lower-emission NG option. The capital investment required for each strategy is also calculated. Details regarding the strategies’ development are in the Supporting Information.

## Results

### Baseline Trends

The baseline run leads to a population-weighted concentration of 0.049 μg/m^3^ (0.16 μg/m^3^ when considering Illinois only) and $1.8–$2.0 B/y in exposure damages, which were computed using the nonlinear concentration–response functions.^1,50^ The range in exposure damages shows the effects of adopting different concentration–response functions and mortality hazard ratios. The linearized exposure damage parameter used in the OPF underestimated the actual damages calculated using the nonlinear concentration–response functions by only 5%–15%. Exposure damages make up about 30% of total costs ($6.3–$6.5 B/y). The remaining 70% represents operational costs ($4.5 B/y).

Several metrics (Table S2**)** were used to evaluate the model’s performance in predicting observed generation trends by comparing 2019 observed and modeled generation levels. The model yields reasonable bias/error values (mean fractional bias (MFB): [−28%–19%]; mean fractional error (MFE): [65%–84%]; *r*^2^: [0.978–0.995]; and root mean square error (RMSE): [24.0–43.8 GWh]) for the 12 months of simulation. The error/bias values reflect the combined impacts of the uncertainty in the cost model and the network constraint transmission parameters. Bias/error values regarding the ISRM have been shown to be well within published air-quality model performance criteria.^8^

Table S4 compares results of the AC OPF run against the DC PF approximation. Accounting for network constraints increases electricity generation by about 4.0%. This is associated with a 4.9% and 3.6% increase in exposure in Illinois and the entire exposure domain, respectively. Neglecting constraints fails to account for $60 M/y (+3.4%) and $160 M/y (+3.7%) in exposure damages and operational costs.

### Exposure-Based OPF

Major exposure reductions are achieved when considering exposure in the OPF. For the all-cost scenario, exposure concentrations (−0.034 μg/m^3^) and damages (−$1.2 to −$1.4 B/y) are reduced by about 70%, but operational costs increase by 6.0% (+$270 M/y). When minimizing exposure damages only, exposure concentrations (−0.038 μg/m^3^) and damages (−$1.3 to −$1.6 B/y) are reduced by 77%, but the increase in operational costs almost doubles (+$540 M/y) relative to the all-cost scenario. The all-cost scenario leads to the lowest total costs ($5.4–$5.4 B/y), as expected, which is a 14%–18% (−$0.89 to −$1.1 B/y) reduction from total baseline costs. The exposure-only scenario still leads to a reduction in total costs relative to baseline conditions (i.e., net benefit), but at a smaller magnitude (−$0.77 to −$1.0 B/y).

**Figure 2:**
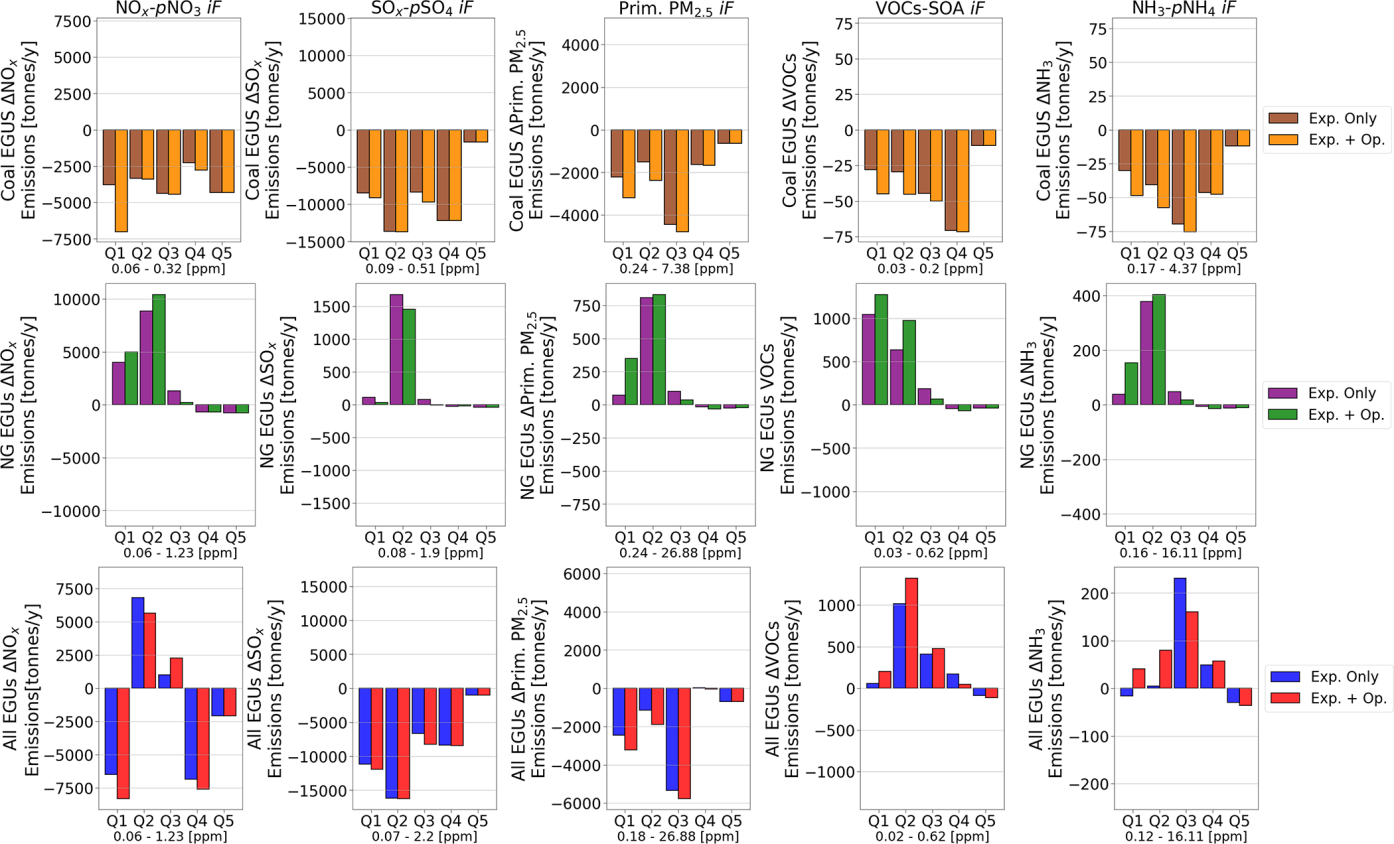
Difference in EGU emissions between the baseline scenario and the two exposure-based optimization scenarios plotted against the intake fraction (*iF*) of the respective EGUs. The modeling framework accounts for both primary and secondary PM_2.5_. Secondary PM_2.5_ precursor emissions that are accounted for are nitrogen oxides (NO_*x*_), sulfur oxides (SO_*x*_), volatile organic compounds (VOCs), and ammonia (NH_3_), which lead to secondary PM_2.5_ species in the form of particulate nitrate (*p*NO_3_), particulate sulfate (*p*SO_4_), secondary organic aerosols (SOA), and particulate ammonium (*p*NH_4_), respectively. EGUs are binned into five quintile groups (from least to greatest *iF*) based on their *iF*s. Each bar represents the net difference in the total sum of emissions of the EGUs, whose *iF* lies within that quintile. The *iF* value range of each pollutant is also shown below each graph. The 1st and 2nd rows correspond to coal and NG-powered EGUs, respectively, while the final row lumps all EGU types together.

Figure 2 plots EGU emission differences between the baseline and two exposure-based scenarios against the *iF*s of the respective EGUs. The 1st and 2nd rows show emissions vs *iF*s for coal- and NG-powered EGUs, respectively, whereas the final row displays emissions for all EGUs. The *iFs* of the EGUs are binned into five quintile (Q1–Q5) groups (from the smallest to the largest *iF*), and each bar represents the total sum of emissions of the EGUs whose *iF* lies within that quintile. Figure 2 provides insights into the two mechanisms that exposure-based optimization uses to mitigate exposure. The first mechanism is reducing emissions from high-polluting EGUs. Row 1 of Figure 2 displays a reduction in coal-powered EGU emissions. Excluding renewable generation, baseline coal-based generation is responsible for 28% of all nonrenewable generation, which is reduced to 6.1% and 1.5% in the all-cost and exposure-only scenarios, respectively. Baseline oil-based generation from peaker plants is responsible for 0.3% of non-renewable generation, which is reduced to negligible levels. Despite their low contribution to generation, oil-powered plants remain a significant target for reduction given that they constitute 4.2% of all exposure. This is attributed to their high emissions and high *iF*s (most peaker plants are located closer to population centers). The mean *iF*s of oil-powered EGUs in this network are 15%–25% higher than the remaining EGUs. Peaker plants and distributed generation units can have *iF*s up to 10 times higher than those of centralized generation plants.^61^ Breakdowns of electricity generation, emissions, and intake contribution by plant types for each optimization scenario are plotted in Figures S2–S7 of the Supporting Information.

To compensate for the reduction in coal- and oil-based generation, the OPF increases generation from NG-powered EGUs. Baseline generation from NG-powered EGUs increases from 15% to 37% and 41% in the all-cost and exposure-only scenarios, respectively, as shown in the 2nd row of Figure 2. However, the OPF safeguards against increased exposure by strategically dispatching low-*iF* NG-powered EGUs. This constitutes the second mechanism the OPF uses to reduce exposure. Despite the 50% increase in exposure attributed to NG-based generation relative to baseline levels, approximately 95% of the emission increase comes from EGUs in Q1–Q2, with the remaining increases coming from EGUs in Q3. No net increase in any pollutant emissions is observed from EGUs in Q4–Q5. Similar trends are observed for both exposure-based optimization scenarios, but the exposure-only scenario achieves higher reductions by reducing emissions from high-polluting plants at a larger magnitude and by transferring a higher proportion of generation from high- to low-*iF* EGUs.

The final row in Figure 2 shows that there is a net reduction in SO_*x*_ and PM_2.5_ emissions in all *iF* quintiles. A net increase is only observed for NO_*x*_ emissions in EGUs that lie in Q2–Q3. There is also some net increase in VOC and NH_3_ emissions due to the increase in NG generation; however, since they contribute to less than 3% of all exposure, this increase is not significant.

### Other PM_2.5_ Exposure Reduction Pathways

#### Post-combustion Emission Control

**Figure 3:**
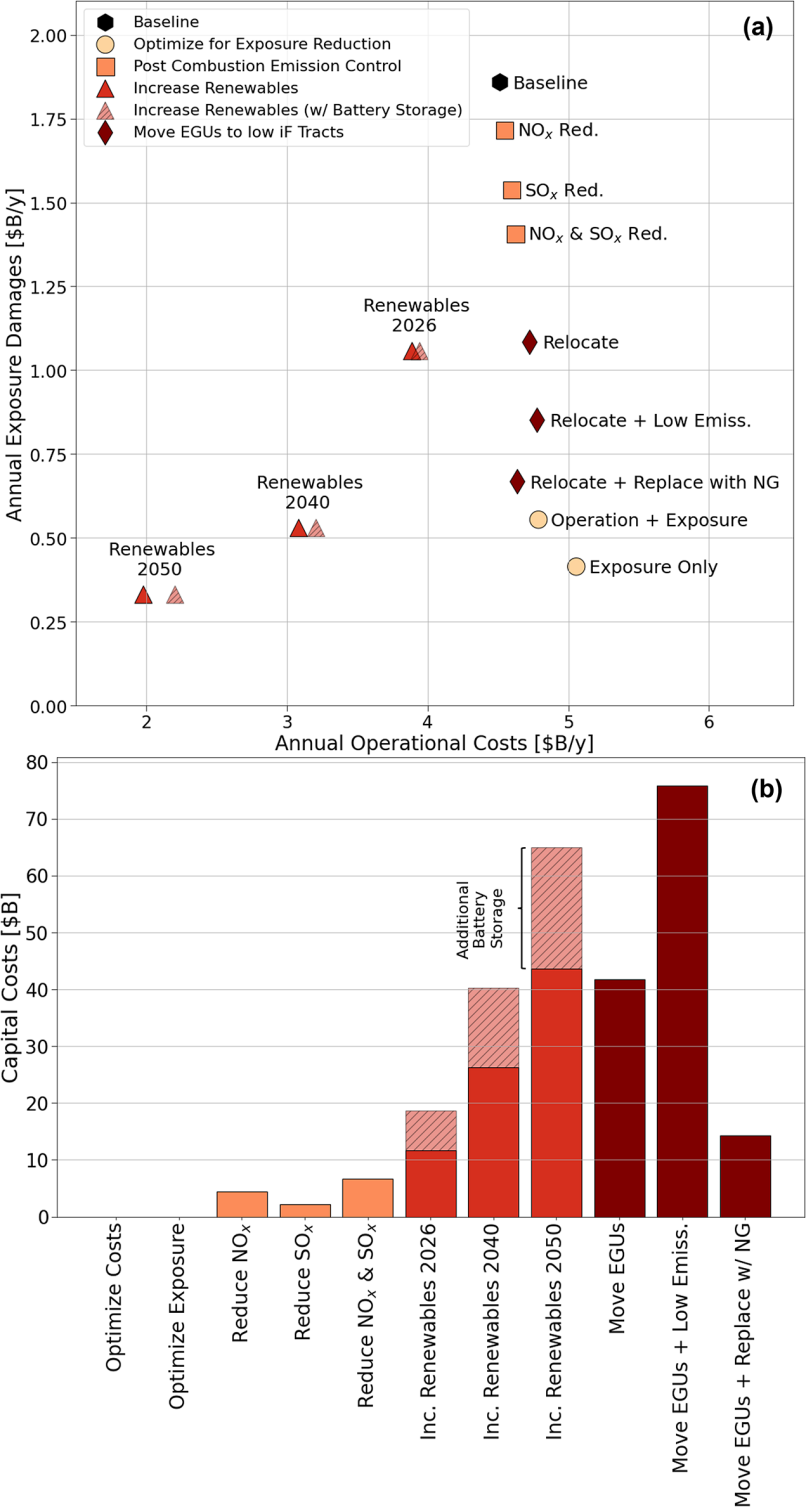
(a) Annual operational costs plotted against annual exposure damages for the baseline AC OPF run and the four exposure reduction strategies explored in the study. Exposure damages shown here represent mid-point values of the range calculated. The renewable energy strategy points with a hatched filling include operation costs with battery storage. (b) Capital costs required for implementing the different exposure reduction strategies. The baseline and two exposure-based optimization reduction strategies do not incur any additional capital costs for their implementation. The hatch portion of the renewable strategies represents the capital costs required for installing battery storage technology in addition to the expanded renewable generation capacity.

Figure 3a plots the annual operational costs against exposure damages of the baseline and mitigation strategies. Retrofitting EGUs with post-combustion emission control technologies leads to the smallest exposure reduction. Emission reductions were applied to NO_*x*_ and SO_*x*_ since they cause 80% of all exposure. 17% is attributable to primary PM_2.5_, with VOCs and NH_3_ emissions responsible for the remaining 3%. Although primary PM_2.5_ constitutes a significant proportion of exposure, 75% of all coal-powered EGUs,^53^ which account for 94% of primary PM_2.5_ emissions and 85% of primary PM_2.5_ intake in the baseline scenario, have electrostatic precipitators (ESPs) or baghouse filters installed to capture particulate matter (PM). Thus, retrofitting the remaining coal-fired plants with PM post-combustion control strategies would only yield marginal reductions in primary PM_2.5_ emissions. Retrofitting eligible EGUs with NO_*x*_ and SO_*x*_ emission control technologies reduces exposure by 8.1% and 16%, respectively. Including both yields a reduction of 23%. Exposure reductions are achieved by lowering NO_*x*_ and SO_*x*_ emissions by 31% and 40%, respectively. Due to the increase in operational and maintenance costs of the EGUs from these technologies, the annual operational costs are raised by 0.76%–2.5%.

#### Relocating High-Polluting EGUs

Relocating high-polluting EGUs to low *iF* zones achieves the next highest exposure reduction. EGUs in the 90th percentile of exposure contributions in the baseline were hypothetically relocated to census tracts in the bottom 10th percentile of *iF* values across all five PM_2.5_ species. All relocated EGUs were coal-powered, except for the one which was oil-powered. Collectively, the relocated EGUs were responsible for 82% of all exposure despite only generating 25% of annual electricity. The mean *iF*s of the tracts that the EGUs were relocated to are 73%–92% lower than the original tracts. If all EGU properties (i.e., emissions, operating costs, and generation capacity) remain the same after relocation, exposure is reduced by 43%. If the EGUs are replaced with advanced low-emitting EGUs of the same plant type or are all replaced with advanced NG, 54% and 62% reduction in exposure can be obtained, respectively. All the low-*iF* tracts were in the southern part of the state, away from densely populated city centers. Figure 1b shows that the high load-demand centers are mostly located in the central and northern parts of the state, meaning that higher transmission losses are expected. The higher transmission losses and operating costs of the advanced plants lead to a 4.6% and 5.8% increase in operational costs if the EGUs were replaced with similar or advanced lower-emitting plants, respectively. Replacing the EGUs with advanced NG-fired plants increases operational costs by just 2.7%.

#### Increasing Renewable Generation

The final strategy involves increasing renewable generation. The 2026 scenario, which assumes 25% renewable generation, still relies on fossil fuel-based generation at 86% of current levels due to the assumed increase of electricity demand. This leads to a 42% reduction in exposure. Increasing renewable generation to Illinois’ 50% renewable generation goal by 2040 and the hypothetical 75% assumed by 2050 still leaves fossil fuel-based generation at 66% and 36% of current levels, respectively. The respective exposure reductions are 70% and 80%. Figure 3a shows that the rate of exposure reductions is diminishing as more renewable generation is added. Initially, a jump from 25% to 50% leads to an additional 28% of exposure reduction, while a similar jump from 50% to 75% only adds an extra 10%. This is because most EGUs that are replaced by renewables initially are operationally expensive coal-powered plants, which contribute the most to exposure. Coal-fired plants in the 90th percentile of exposure contribution cause about 80% of all exposure. Thus, replacing high-polluting EGUs first would lead to a major initial reduction in exposure, but the reduction rate will begin to diminish as renewable generation increases since the remaining EGUs in line to be replaced are “cleaner”. Increasing renewables is the only strategy that leads to a reduction in annual operational costs (14%–56%). Including battery storage reduces the magnitude of reduction in operating costs by 2%–6% relative to no storage. Table S3 summarizes the exposure and cost metrics computed for the baseline run and all strategies.

### Capital Costs of Mitigation Strategies

Figure 3b shows the capital costs associated with each strategy. Exposure optimization requires no additional capital costs since the OPF dispatches currently available EGUs. Emission control strategies have the lowest capital costs ($2.2–$6.7 B). Relocating high-polluting EGUs leads to varying capital costs depending on what the replacements are. Replacing them with lower-emitting NG plants costs around $14 B, but choosing their current types or advanced lower-emitting versions will lead to higher capital costs ($42–$76 B). This is due to capital costs of coal-powered EGUs becoming higher than those of NG-powered EGUs recently.^59^ The capital investment in associated transmission infrastructure (i.e., transmission lines and transformers) for the new EGUs is much smaller (∼$300 M). Increasing renewable generation with no battery storage requires $12 B, $26 B, and $43 B in capital investments for the 2026, 2040, and 2050 scenarios, respectively, which is 30%–40% lower than if battery storage was included. A combined total cost for all strategies is calculated by obtaining an equivalent annual cost for the strategies’ capital investments with varying discount rates and expected lifetimes of the investments. These are then added to the annual operation dispatch costs and annual exposure damages. Results of the analysis are plotted in Figures S7–S10 in the Supporting Information. The choice of the discount rate and the average expected lifetime of the capital investment greatly affects whether a strategy would lead to a net benefit (i.e., lower total costs compared to the baseline). No strategies yield a reduction in combined costs relative to the baseline when discount rates are assumed to be high (e.g., 7%–9%) and expected capital investment lifetimes are low (e.g., 10–20 years), with the exception of the exposure optimization scenarios since they do not involve any capital costs. If the assumed discount rate is low (e.g., 3%–5%) but the investment’s capital lifetime also remains low, a few strategies apart from the exposure optimization strategies lead to a reduction in combined costs. If discount rates are assumed to be low with high capital investment lifetimes (e.g., 30–40 years), all strategies yield a reduction in combined costs except when EGUs are relocated and are not replaced with NGs instead of their original coal types.

## Limitations and Uncertainty Assessment

Limitations in the data sources and the modeling framework introduce uncertainties to the study. In terms of load demand, given that only representative average daily simulations of each month are used, the results may not capture extreme peak day loads. Extreme daily peaks may be responsible for higher emissions and thus higher exposure, especially if high emission/*iF* plants are dispatched. Another limitation with load modeling is that only the temporal variation in load is captured, whereas the spatial distribution of load weights within the network is assumed to be fixed.

The OPF framework is rooted in the assumption that system operators dispatch EGUs in a manner that minimizes dispatch costs. Thus, the choice in the cost model will dictate the dispatch schedule. While effort was made to incorporate a comprehensive cost model and include EGU-specific costs when possible, there are certain dispatch decisions that may be independent of cost and cannot be reflected in this type of framework. EGU costs and capacities are calibrated based on historical generation trends to attempt to capture those hidden variables, but that practice may introduce other types of errors, especially given that past generation trends may not be reflective of future ones. The model constraints also account for dispatching decisions that reflect other operator goals, such as ensuring system stability and safety. However, large uncertainties exist in the modeled parameterized system components, and they may not be reflective of the actual network parameters in place. In some instances, certain branch constraints had to be relaxed to ensure that the AC power flow converged. Regardless, the goal of this exercise is to demonstrate the usefulness of incorporating such constraints and showcase the benefits of the model in the hands of a system operator who possesses all necessary data.

Since average EGU emission factors (when EGU-specific ones cannot be obtained) and linearized emissions-concentration relationships are used, there may be some inaccuracy in modeling EGU emissions and subsequent concentrations. An added limitation is that EGU per-unit monetized damages [$ per MWh] developed reflect annually averaged changes in PM_2.5_ concentrations and not actual instantaneous changes in concentration that would be incurred due to those EGU emissions. However, average exposure trends are satisfactory since health impacts are dictated by long-term exposure to PM_2.5._^3,4^

Despite several sources of uncertainty in the data and the methodology, the bias/error metrics calculated in Table S2 show that the OPF predicts observed generation reasonably well. These bias/error values reflect the combined impacts of the uncertainty in the cost model and the network constraint transmission parameters. Reasonable bias/error values mean that the approximated network transmission parameters do not activate constraints that impede the EGUs from dispatching power at their observed levels. This provides confidence in the EGU generation levels used to develop the cost and exposure profiles. InMAP’s proven performance in predicting PM_2.5_ concentrations also provides confidence that the obtained exposure results are within an acceptable range of error.

The exposure results do not veer far from results found in similar studies. The baseline Illinois population-weighted PM_2.5_ concentration is 0.16 μg/m^3^, which is broadly in line with that calculated for California EGUs (0.06 μg/m^3^).^62^ Discrepancies in the results can be attributed to differences in EGU plant types, where Illinois has a much higher generation from coal-powered EGUs and lower renewables. The modeled exposure damages in Illinois ($1.0 B/y) are also about 50% lower than those in Thind et al.^16^ ($2.3 B/y). The discrepancy may be due to the difference in years modeled (2014 vs 2019), where past years may have had more fossil-based generation and less renewables. It could also be due to the difference in the concentration–response function and the mortality hazard ratio used. When adjusting the hazard ratio in our exposure damages function to the range of possible values, the damages range between $1.0 and $2.4 B/y.^1,3,4^ Sufficient agreement with both the generation and the exposure results shows that the predicted baseline damages and the potential reductions are reasonable and attainable.

## Discussion

This research has developed an OPF model that can be used by system operators for long-term capacity planning and minute-by-minute adjustment of generator dispatch while accounting for PM_2.5_ exposure damages from EGUs. The contributions of this work are in developing a model that can inform decision-making for managing internal operations and external impacts of electric power systems. The use of an OPF model, as opposed to other dispatch models, ensures the feasibility of proposed mitigation strategies under present network setups while also considering other operating goals such as system stability and reliability. Modeling baseline exposure trends and the mitigation potential of strategies through an OPF can aid policymakers in mandating systemwide changes to minimize health and climate damages while balancing network operating parameters such as dispatch costs.

The results demonstrate how system operators can explore pathways to mitigate exposure under realistic network conditions and while respecting network constraints. Although ED models can use historic EGU generation data to internalize the additional generation from transmission losses, projecting future generation trends while accounting for transmission losses and constraints can only be achieved with an OPF model. Neglecting transmission constraints fails to account for up to $60 M/y in exposure damages and $240 M/y in operational costs (∼4%).

Another aspect of the analysis is showing the extent of exposure damage reduction possible when internalizing the externality of exposure into the dispatch cost model. Baseline exposure damages are on the order of $1.9 B/y, more than half of which occur across state lines. Accounting for exposure damages in the OPF results in a 70% reduction in damages relative to baseline conditions, which is a reduction magnitude in accordance with previous studies minimizing EGU-related damages from PM_2.5_ exposure (e.g., 50%,^17^ 11%–66%,^22^ 60%–90%,^20^ 80%^19^). Reductions are achieved by dispatching low-emission EGUs located in low-*iF* zones. While accounting for exposure damages only in the OPF objective function achieves an additional 7% of reductions, the increase in operational costs almost doubles. In both cases, the exposure damage reductions outweigh the increase in operational costs, leading to net benefits. The relative increase in operational costs (6.0%–12%) to reduce PM_2.5_ exposure is also in line with mitigation cost findings in similar studies (0.10%–5.8%,^22^ 14%^20^). An added benefit of the exposure optimization strategy is that the reductions come at no additional capital investment while still achieving equal or higher reductions relative to other strategies.

Retrofitting EGUs with the best-available emission control technology incurs the smallest capital costs but also leads to the smallest exposure reduction since most EGUs are already equipped with some form of emission control technology. EGU emissions should not be the sole dimension to consider when mitigating exposure since EGUs with high *iF*s may discount the emission reduction benefits. Instead, both emissions and EGU *iF*s need to be targeted with actions such as relocating high exposure-inducing EGUs to low-*iF* zones. Almost 80% of all exposure in this study is attributed to EGUs fulfilling only 25% of all electricity demand. Had these EGUs been built in the low-*iF* zones that they were hypothetically relocated to, about 43% of all exposure would have been avoided. Given that most relocated EGUs were coal-powered, replacing them with lower-emitting, NG-based EGUs reduces exposure by an additional 20% and lowers the operational and capital costs. Eliminating emissions entirely through renewable generation achieves the highest magnitude of exposure and operational cost reductions. Interestingly, the initial increase in renewable generation is what yields the highest benefit to exposure reduction since it eliminates emissions from the highest exposure-inducing and operationally expensive EGUs (i.e., high-*iF* coal-fired plants).

While renewable generation may be the best option in the long-term, the high capital investment and Renewable Portfolio Standard (RPS) implementation timelines may hinder near-term exposure reduction goals. Another issue related to renewables is that of intermittency, which could be tackled by battery storage in the long run when that technology scales and becomes more affordable; however, readily dispatchable EGUs will still be required in the short term. Figure S3 in the Supporting Information shows that exposure from secondary PM_2.5_ due to precursor NO_*x*_ emissions from NG plants can still yield non-negligible health exposure impacts even in a scenario where exposure damages are minimized. It is expected that NG-based EGUs will likely be the predominant source of baseload power in the near future. The relocation strategy results show that where EGUs are sited, not just their plant types, can play a crucial role in dictating the amount of exposure damages that may take place. Thus, strategic siting of any potential new fossil-based generation in low-*iF* zones should remain an important consideration since it could limit damages significantly. Even though relocating and/or dispatching low-*iF* EGUs may significantly reduce exposure damages, it still leaves the issue of GHG emissions, whose impacts are independent of the emission location. Co-optimizing for health and climate goals can increase their benefits relative to optimizing for those objectives separately.^19,20^ From a policy perspective, some combination of different strategies would be the best path forward for exposure reduction. Running multiple strategies collectively while also accounting for exposure damages in the OPF objective function achieves reductions upward of 90%. The significance of this combined approach for mitigating exposure becomes even more critical in emerging regions where electricity demand is expected to increase in the upcoming decades. Excess mortality attributable to power generation in Southeast Asia is expected to rise by 161% between 2025 and 2040 (from 26,500 annual deaths in 2025 to 69,200 annual deaths in 2040),^63^ meaning that active planning of the type of EGUs that are built, their location, and the manner in which they are dispatched is essential for mitigating future exposure impacts.

Future work should focus on analyzing the effects of such strategies on network flow and stability. A more thorough investigation of the effects of adapting power networks to meet climate and health goals should be undertaken when actual network data become available. The model should also be expanded and applied to a network size at the ISO level, which is the level where high-level dispatch decisions take place. Additionally, given that the model is solved to optimality over 1 h time horizons, future iterations of this work could focus on making the model more comprehensive by including elements such as a multi-period dynamic OPF with ramping, minimum generation, and unit commitment constraints, as well as stochastic renewable generation or energy storage components. The resolution at which the model is solved could be increased, where an actual simulation of all hours of the year instead of averaged 24 h periods duplicated for every day of the month could better capture the impacts of peak loads on operation costs and exposure. Consideration should also be made toward accounting for the additional emissions that are incurred from the extra cycling (i.e., extra plant start-ups and ramping) that fossil-based plants would undergo in scenarios with high renewable generation, which would likely discount their benefits slightly.^64^ The current all-cost scenario weights operation costs and exposure damages equally, which assumes that they are comparable. Policy makers may have varying opinions about how the different cost metrics should be compared,^65^ where some might suggest that exposure damages need to be weighted more heavily, while others may argue that system operators need not fully internalize the externality of exposure and that it be shared with the EGU operators. Future work should focus on establishing policy mechanisms on how exposure damages should be weighted and how the exposure externalities of electricity generation could be internalized within this EGU dispatching framework. Next steps should also incorporate equity considerations into the reduction strategies. Previous studies^17,19,66,67^ have used optimization techniques to assess and reduce the exposure disparity for disadvantaged groups, and the OPF framework can be augmented with such capabilities. Equity consideration should be incorporated into exposure mitigation plans of all infrastructure to ensure that reducing absolute exposure does not come at the expense of increasing the exposure disparity for disadvantaged groups.^16,19,50,67−70^

## Supporting Information Available

AC OPF formulation, information regarding network development, information regarding the development of the different strategies, information regarding the development of the exposure damages factor, map of the exposure domain, electricity generation and emission metrics by EGU plant types, total annualized costs of the different scenarios and strategies, and result data tables (PDF)
