# Supplementary material for: Exploring Regional Fine Particulate Matter (PM2.5) Exposure Reduction Pathways Using an Optimal Power Flow Model: The Case of the Illinois Power Grid
Source: Environ Sci Technol. 2023 May 16;57(21):7989–8001. doi: 10.1021/acs.est.2c08698 (PMC10233793; doi:10.1021/acs.est.2c08698)
Supplement: Supplementary file 1 — es2c08698_si_001.pdf [file es2c08698_si_001.pdf]

Exploring Regional Fine Particulate Matter ($PM_{2.5}$) Exposure Reduction Pathways using an Optimal Power Flow Model: The Case of the Illinois Power Grid

(Supplementary Information)

Ahmad Bin Thaneya[†*] *and Arpad Horvath*[†]

[†]Department of Civil and Environmental Engineering, University of California, Berkeley, California, 94720, United States

Pages: 37

Figures: 10

Tables: 4

1. AC Optimal Power Flow Formulation

Network Equations

A brief background for the optimal power flow (OPF) formulation will be provided here. The purpose behind this background is to provide those who may not be entirely familiar with OPF models with an accessible introduction to their fundamental theoretical components. For a more detailed overview, interested readers are referred to Frank & Rebbenack,[1] which this formulation adopted from. **Table S1** summarizes the model's variables and parameters.

Mathematical models are used for the formal and systematic representation of power systems as well as for solving the OPF problem. A general setup of the OPF problem and necessary background will be presented here. The most common representation of power systems is through an undirected graph. Within this study, a general power systems network will be represented by $\mathrm{H} = (N, A)$, where N represents the set of nodes (i.e., buses) and A represents the set of edges within the network. Each edge represents a $branch\ (i, k)$ that connects an upstream $bus\ (i)$ to a downstream $bus\ (k)$. Branches can be physical representations of different power systems equipment such as lines or transformers. Relevant $branch\ (i, k)$ parameters that characterize power systems equipment include the impedance ($\mathbb{Z}_{ik}$), which is broken up into $line\ resistance\ (R_{ik})$ and $line\ reactance\ (X_{ik})$ as per **(1).** Within this notation, roman letters (e.g., $\mathbb{Z}, \mathbb{V}, \mathbb{I}, \mathbb{Y}$) will be used to signify phasor quantities.

$$\mathbb{Z}_{ik} = R_{ik} + jX_{ik} \qquad \mathbf{(1)}$$

The current on a line is generally represented by ($\mathbb{I}$). This can be used to represent the general form of Ohm's law to calculate the voltage drop ($\mathbb{V}$) across a circuit element, which is shown in **(2)**. In this general formulation

$$\mathbb{V} = \mathbb{I}\mathbb{Z} \qquad (\mathbf{2})$$

Therefore, the current leaving (or injected into) bus_i ($\mathbb{I}_i$) can be represented using **(3).**

$$\mathbb{I}_i = \frac{\mathbb{V}_{ik}}{\mathbb{Z}_{ik}} \qquad (\mathbf{3})$$

In power flow problems, it is often more convenient to replace the impedance ($\mathbb{Z}_{ik}$) of a line by its admittance ($\mathbb{Y}_{ik}$) as per **(4).**

$$\mathbb{Y}_{ik} = \frac{1}{\mathbb{Z}_{ik}} = G_{ik} + jB_{ik} \qquad (\mathbf{4})$$

where (G_{ik}) represents the $branch\ conductance$ and (B_{ik}) represents the $branch\ susceptance$. Therefore, Ohm's law can be rewritten as shown in **Equation (5).**

$$\mathbb{I}_i = \mathbb{Y}_{ik}\mathbb{V}_{ik} \qquad (\mathbf{5})$$

This allows one to write Ohm's law for an entire network, and it can be represented compactly in matrix notation as per **(6)**, where $\bar{\mathbb{I}} = (\mathbb{I}_1, \ldots, \mathbb{I}_N)$ is the vector of phasor source currents that are injected into each bus, $\bar{\mathbb{V}} = (\bar{\mathbb{V}}_1, \ldots, \bar{\mathbb{V}}_n)$ is the phasor bus voltage vector, and ($\bar{\bar{\mathbb{Y}}}$) is the complex bus admittance matrix.

$$\bar{\mathbb{I}} = \bar{\bar{\mathbb{Y}}}\bar{\mathbb{V}} \qquad (\mathbf{6})$$

Power Flow Equations

The power flow problem is a feasibility problem that seeks to determine a solution to the network equations, without a specific objective function. The power flow equations are thus used as constraints in the OPF formulation to ensure that the optimal solution to the OPF problem is a

feasible one. To derive the power flow equations, we begin by assuming that each node within the network will represent a bus where power can be generated or consumed. Each bus_i $(i \in N)$ will be characterized by the following four variables:

1. Voltage magnitude: $|V_i|$
2. Voltage phase angle: δ_i
3. Net real power injection: P_i
4. Net reactive power injection: Q_i

Solving the power flow problem requires developing a relationship between all four variables at all points within a network. Doing so requires developing a system of power flow equations for the network which will have the form shown in **(7)** and **(8)**. The final form of the equations will be shown in **(14)** and **(15)** after they are derived in this section. Equations **(7)** and **(8)** represent power injections into each bus_i, which also equate to the difference between power generation (P_i^G, Q_i^G) and power load (P_i^L, Q_i^L) at each bus.

$$P_i(|V|, \delta) = P_i^G - P_i^L \qquad \forall\, i \in N \quad (\mathbf{7})$$

$$Q_i(|V|, \delta) = Q_i^G - Q_i^L \qquad \forall\, i \in N \quad (\mathbf{8})$$

Given that we have two governing power flow equations and four variables to solve for, a deterministic solution requires fixing two of each of the four variables at each bus. Three types of buses can be found in the network (depending on what variables are specified and what variables must be solved for at the bus). Setting up the network in such a manner allows for an equal number of unknowns and equations, thus allowing for a deterministic feasible solution. The three types of buses are as follows:

1. Load Bus (P-Q bus): These represent buses where the load is known and fixed, while the voltage magnitude and phase angle is solved for.
    a. Specify: P_i and Q_i
    b. Solve For: $|V_i|$ and δ_i

2. Generator Bus (P-V bus): The real power injections and voltage magnitudes within these buses are known, while the voltage phase angle and reactive power injections is solved for. This represents allowing a local source of reactive power to regulate the bus voltage.
    a. Fix: $|V_i|$ and P_i
    b. Solve For: δ_i and Q_i

3. Slack Bus (δ-V bus) [typically used as the reference bus and given index $i = 1$]. Here, the voltage magnitude and phase angle are fixed while the power injections are solved for. Fixing the voltage magnitude and phase angle also allows for the remaining network bus voltages and phase angles to be determined. Each network must have one slack bus where the power injections are solved for such that power flow equations have a feasible guaranteed solution.
    a. Fix: $|V_1| = 1.0$ p.u. and $\delta_1 = 0°$
    b. Solve For: P_1 and Q_1

Next, we move towards specifying the exact form of equations **(7)** and **(8)**. This is achieved by transforming the current flows within the network into power flows. Using the network equations **(5)** and **(6)**, one can find that the current being injected into bus_i can be represented as follows:

$$\mathbb{I}_i = \sum_{k \in N} \widetilde{\mathbb{Y}}_{ik} \mathbb{V}_k \qquad \mathbf{(9)}$$

Noting that **Equation (10)** shows complex power injected into bus k,

$$\mathbb{S}_i = P_i + jQ_i = \mathbb{V}_i \mathbb{I}_i^* = \mathbb{V}_i \left[\sum_{k \in N} \widetilde{\mathbb{Y}}_{ik} \mathbb{V}_k \right]^* \qquad \mathbf{(10)}$$

and defining the voltage ($\mathbb{V}_k$) and negative sum of admittances of the branch connecting bus_i and bus_k ($\widetilde{\mathbb{Y}}_{ik}$) using **(11)** and **(12)**, respectively, where (δ_k) is the voltage angle at bus_k, (θ_{ik})

is the current phase angle associated with the admittance between bus_i and bus_n, we can write the apparent power ($\mathbb{S}_k$) at bus_i using **(13)**.

$$\mathbb{V}_k = |V_k| e^{j\delta_k} \qquad \mathbf{(11)}$$

$$\widetilde{\mathbb{Y}}_{ik} = |\tilde{Y}_{ik}| e^{j\theta_{ik}} = G_{ik} + jB_{ik} \qquad \mathbf{(12)}$$

$$\mathbb{S}_i = P_i + jQ_i = |V_i| \sum_{k \in N} |\tilde{Y}_{ik}| |V_k| e^{j(\delta_i - \delta_n - \theta_{ik})} \qquad \mathbf{(13)}$$

The equations can also be written in rectangular form by taking the real (**14)** and imaginary parts (**15)**, separately. These represent the full form of the power flow equations that will be used as constraints in the OPF to ensure the feasibility of the OPF solution.

$$P_i(|V|, \delta) = \sum_{k \in N} |V_i||V_k| [G_{ik} \cos(\delta_i - \delta_k) + B_{ik} \sin(\delta_i - \delta_k)] = P_i^G - P_i^L \qquad \forall\, i \in N \qquad \mathbf{(14)}$$

$$Q_i(|V|, \delta) = \sum_{k \in N} |V_i||V_k| [G_{ik} \sin(\delta_i - \delta_k) - B_{ik} \cos(\delta_i - \delta_k)] = Q_i^G - Q_i^L \qquad \forall\, i \in N \qquad \mathbf{(15)}$$

AC Optimal Power Flow

The OPF can be formulated as an optimization problem when the power flow equations derived in **(14)** and **(15)** are combined with an objective function. Objective functions of OPF problem typically minimize total costs of electricity generation while also ensuring that the system is operating within safety limits. The optimization variables within the OPF formulation are the real power injections at generator buses ($\Gamma \subseteq N$), while voltage magnitudes and angles are dependent state variables used to formulate the constraints. The AC OPF problem for a specific time interval (e.g., 1-hour) can be formulated as shown in **(16)**.

$$\min_{P_i^G} \sum_{i\in\Gamma} f_i(P_i^G)$$

s.t.

$$P_i(|V|,\delta) = P_i^G - P_i^L \qquad \forall\, i \in N$$

$$Q_i(|V|,\delta) = Q_i^G - Q_i^L \qquad \forall\, i \in N$$

$$P_i^{G,min} \le P_i^G \le P_i^{G,max} \qquad \forall i \in \Gamma \tag{16}$$

$$Q_i^{G,min} \le Q_i^G \le Q_i^{G,max} \qquad \forall i \in \Gamma$$

$$(|V_i|cos\delta_i - |V_k|cos\delta_k)^2 + (|V_i|sin\delta_i - |V_k|sin\delta_k)^2 \le \frac{(I_{ik}^{max})^2}{\left|\tilde{Y}_{ik}\right|^2} \qquad \forall(i,k) \in A$$

$$\left|V_i^{min}\right| \le |V_i| \le |V_i^{max}| \qquad \forall i \in N$$

$$\delta_i^{min} \le \delta_i \le \delta_i^{max} \qquad \forall i \in N$$

Where:

$P_{i,i\in G}^G$ is the power produced by the *i*th generator and represents the optimization variable of the model.

$f_i(P_i^G)$ is the cost to operate unit *i* at the output level P_i^G.

The OPF will minimize system costs by controlling the power produced at all the generators subject to the network constraints. The constraints can be interpreted as follows:

- The first two equality constraints denote the power flow equations derived in the previous subsection that must be satisfied. AC power flow equations must be satisfied by the converged to solution in order for it to be a feasible operating point of the power

network. The load real (P_i^L) and reactive power (Q_i^L) are given and fixed for a specific time-period (i.e., 1-hr time interval in this instance).

- The third constraint represents the real power limits at the buses. ($P_i^{G,min}$) is usually set as 0 unless a generator has a fixed minimum generation requirement. Renewable sources are set to their historic hourly output by fixing their real power generation limits.
- The fourth constraint represents the reactive power limits at the buses. Reactive power loads and minimum and maximum limits are based on power factor data approximated from other synthetic networks.[2–4]
- The fifth constraint represents the branch limits, which sets an upper limit on the magnitude of the current that can pass by any branch within the network. This constraint is derived using Ohm's law, where branch current is dependent on the voltages between the branch and the branch admittance. Max loading is also based on parametrized line data.[2–5] The voltages in the constraint are expanded and written in rectangular form to match the rest of the formulation, but it can be compacted and written in phasor form as follows:

$$\left|\tilde{Y}_{ik}\right||(\mathbb{V}_i - \mathbb{V}_k)| \leq I_{ik}^{max} \qquad \forall(i,k) \in A \qquad \textbf{(17)}$$

- The sixth constraint represents the maximum and minimum allowable bus voltage magnitude limits. For this formulation, $\left|V_i^{min}\right|$ is set to 0.98 p.u. while $|V_i^{max}|$ is set to 1.04 p.u.
- The seventh constraint represents the maximum and minimum allowable bus voltage angle limits. For this formulation, δ_i^{min} is set to $-45°$ while δ_i^{max} is set to $+45°$.

DC Optimal Power Flow

To quantify the additional costs and damages that arise from accounting for network constraints and reactive power flow, we employ a DC power flow approximation to the network. A DC power flow approximation is a linearized version of the AC power flow equations. The following assumptions hold for the DC power flow approximation:

- There is no reactive power flow on the network.
- The transmission system is assumed to be lossless with branch resistances ($R_{ik} = 0$). This leads to all phase angles $\theta_{ik} = \pm 90°$ and branch conductances ($G_{ik} = 0$).
- Differences between adjacent voltage angles are small leading to $\sin(\delta_i - \delta_k) \approx \delta_i - \delta_k$ and $\cos(\delta_i - \delta_k) \approx 1$.
- All bus voltages in the network are at unity ($|V_i| \approx 1.0$).

Applying these assumptions to **equation (15)** leads to the following approximation for real power transfer:

$$P_i^{DC}(\delta) \approx \sum_{k \in N} B_{in}(\delta_i - \delta_k) = P_i^G - P_i^L \qquad \forall\, i \in N \qquad \mathbf{(18)}$$

Thus, the DC approximate optimization program is as follows:

$$\min_{P_i^G} \sum_{i \in \Gamma} f_i(P_i^G)$$

s.t.

$$P_i^{DC}(\delta) = P_i^G - P_i^L \qquad \forall\, i \in N \tag{19}$$

$$P_i^{G,min} \le P_i^G \le P_i^{G,max} \qquad \forall i \in \Gamma$$

$$\delta_i^{min} \le \delta_i \le \delta_i^{max} \qquad \forall i \in N$$

Table S1 Summary of power systems network notation used and analysis variables and parameters.

Symbol	Description	Units
**H**	Power Systems network graph	[-]
$\boldsymbol{N}$	Set of nodes (i.e., buses)	[-]
$\boldsymbol{A}$	Set of edges (i.e., power transmission equipment)	[-]
$\boldsymbol{\Gamma}$	Set of generators (i.e., electricity generation units)	[-]
$\mathbb{I}_i$	Current leaving (or injected into) $\boldsymbol{bus_i}$	[A]
$\mathbb{V}_i$	Voltage at $\boldsymbol{bus_i}$	[kV / p.u.]
$\mathbb{Z}_{ik}$	Impedance of $\boldsymbol{branch_{ik}}$	[Ω]
$\boldsymbol{R_{ik}}$	Resistance of $\boldsymbol{branch_{ik}}$	[Ω]
$\boldsymbol{X_{ik}}$	Reactance of $\boldsymbol{branch_{ik}}$	[Ω]
$\mathbb{Y}_{ik}$	Admittance of $\boldsymbol{branch_{ik}}$	[Ω^{-1}]
$\widetilde{\mathbb{Y}}_{ik}$	Negative sum of admittances of the branch connecting $\boldsymbol{bus_i}$ and $\boldsymbol{bus_k}$	[Ω^{-1}]
$\boldsymbol{G_{ik}}$	Conductance of $\boldsymbol{branch_{ik}}$	[Ω^{-1}]
$\boldsymbol{B_{ik}}$	Susceptance of $\boldsymbol{branch_{ik}}$	[Ω^{-1}]
$\bar{\mathbb{I}}$	Vector of source currents injected into the network buses	[A]
$\bar{\mathbb{V}}$	Bus voltage vector	[kV / p.u.]
$\mathbb{Y}$	Bus admittance matrix	[Ω^{-1}]
$\lvert\boldsymbol{V_i}\rvert$	Voltage magnitude at $\boldsymbol{bus_i}$	[kV / p.u.]
$\boldsymbol{\delta_i}$	Voltage phase angle at $\boldsymbol{bus_i}$	[degrees]
$\boldsymbol{P_i}$	Net real power consumed or generated at $\boldsymbol{bus_i}$	[MW]
$\boldsymbol{Q_i}$	Net reactive power consumed or generated at $\boldsymbol{bus_i}$	[MVar]
$\boldsymbol{P_i^G}, \boldsymbol{Q_i^G}$	Real and reactive power generation at $\boldsymbol{bus_i}$	[MW/MVar]
$\boldsymbol{P_i^L}, \boldsymbol{Q_i^L}$	Real and reactive power load at $\boldsymbol{bus_i}$	[MW/MVar]

$\mathbb{S}_k$	Net apparent power at $\boldsymbol{bus_k}$	[MVA]
$\boldsymbol{\theta_{ik}}$	Current phase angle associated with the admittance between $\boldsymbol{bus_i}$ and $\boldsymbol{bus_k}$	[degrees]
$\boldsymbol{f_i(P_i^G)}$	Cost function (i.e., objective function) to operate $\boldsymbol{generator_i}$ at output load $\boldsymbol{P_i^G}$	[$/hr]
$\boldsymbol{I_{ik}^{max}}$	Loading limit on $\boldsymbol{branch_{ik}}$	[A]
$\boldsymbol{P_i^{G,min}, P_i^{G,max}}$	Real power minimum generation and capacity generation at $\boldsymbol{bus_i}$	[MW]
$\boldsymbol{Q_i^{G,min}, Q_i^{G,max}}$	Reactive power limits at $\boldsymbol{bus_i}$	[MVar]
$\boldsymbol{\lvert V_i^{min}\rvert, \lvert V_i^{max}\rvert}$	Voltage magnitude limits at $\boldsymbol{bus_i}$	[kV]
$\boldsymbol{\delta_i^{min}, \delta_i^{max}}$	Voltage angle limits at $\boldsymbol{bus_i}$	[degrees]
$\boldsymbol{C_i}$	Unit operation (including maintenance & fuel) unit cost of $\boldsymbol{generator_i}$	[$/MWh]
$\boldsymbol{U}$	Set of pollutants considered (Primary $PM_{2.5}$/Secondary $PM_{2.5}$ from Emission Precursors)	[-]
$\boldsymbol{M}$	Set of exposure zones within the study area.	[-]
$\boldsymbol{E_{iu}}$	Emission factor of pollutant $\boldsymbol{u}$ for $\boldsymbol{generator_i}$	[g/MWh]
$\boldsymbol{\Lambda}$	InMAP generated population augmented source-receptor (intake fraction) matrices for Primary (Secondary) $PM_{2.5}$ (Precursors)	[kg$PM_{2.5}$ inhaled per hour per g/h of pollutant emitted]
$\boldsymbol{\nu}$	Linearized exposure factor quantifying damages from unit of pollutant intake	[$ per kg$PM_{2.5}$ inhaled]
$\boldsymbol{D_i(P_i^G)}$	Exposure Damages from operating $\boldsymbol{generator_i}$ at output load $\boldsymbol{P_i^G}$	[$/hr]

2. Network Development

Details regarding network development, assumptions, and data sources are provided below.

Transmission Line and Substation Data

Transmission line and substation data are obtained from the Homeland Infrastructure Foundation-Level Data (HIFLD) database.[6,7] Substations within the network represent nodes where transmission lines connect as well as equipment that switch, transform, and regulate electric power. HIFLD only holds complete data for substations rated at 69 kV or above, meaning these substations regulate power flow at the transmission level (i.e., a step above the distribution level). Transmission lines from HIFLD are also rated by voltage class and range between 69 kV and 765 kV. Some data cleaning has been carried out to ensure that there is no mismatch between the subsequent transmission line voltage ratings as well as the voltage ratings of the substations they connect to. Since transformer data is not available, transformers are parametrized and added to substations that connect transmission lines with different voltage ratings. It is assumed that these transformers step voltage up and down between those lines. Transformer and transmission line parametrization is carried out based on outlined methods for developing synthetic power networks.[2,4,5,8]

Load Demand Data

Running an OPF requires knowing the spatial distribution of load within the network. To our knowledge, the exact distribution of network load is not publicly available. Therefore, several steps are taken to obtain a representative, spatially and temporally resolved load profile for this network. It is assumed that power load demand across the network is consumed at the substation level, specifically at substations where voltage levels are stepped down to distribution level

voltages. Substations which meet that voltage criteria are identified and are designated as load buses. A load weight is then assigned to each of the load buses. The load weight represents the percent of total load in the network consumed at that bus. It is quantified based on the location of the load buses within the network with respect to Illinois' Electric Retail Service Territories.[9] Each load bus is assigned to the Retail Service Territory(ies) it lies within. A load weight for each territory is developed based on the ratio of its total electric sales relative to the total electricity sales of all territories within Illinois. It is assumed that the utilities that lie within Illinois' Retail Service Territories, in addition to the external balancing grid, meet all the electric load demand in Illinois. The load weight is then equally distributed amongst all load buses within each territory. Since certain load buses lie within two or more territories, their respective load weights from each territory are summed to obtain a total load weight for each bus. **Figure 1(b)** in the main text shows the distribution of load weights to the network substations.

The next step involves obtaining the total annual electric energy demand for the state of Illinois. Specifically, 2019 annual electric energy consumption data is used.[10,11] The temporal variation in total system load demand for each 24-hr period of our simulation was generated based on hourly load data obtained from Midcontinent Independent System Operator (MISO), which most of the state's power utilities are operated by.[12] Once the total hourly load for the entire network is known, it is then spatially distributed to the load buses based on their corresponding load weights. The load buses are also augmented with reactive power data based on power factor data approximated from other synthetic networks.[2,4,8]

EGU Data

EGU data are obtained from publicly available eGRID2019.[10] Each EGU is designated as a generator bus and is then connected to the network based on its location. Since voltage ratings of

EGUs are typically in the 10s of kV range, each EGU is connected to a transformer to step its voltages up to transmission level voltages. EGU plant type and nameplate capacity are also obtained from eGRID2019. EGU reactive power limits are approximated following methods in developing synthetic networks. [2,4,8] Temporally resolved plant specific operation, maintenance, and fuel costs [$/MWh] are used when data is available, but average costs based on plant type are used otherwise. EGU costs and capacity constraints are also calibrated based on the historic generation of each EGU. Since some of the average costs accounted for in the modeling may not be inclusive of all costs that may affect the choice or dispatching sequence, we calibrate the per-unit costs of historically high generating EGUs by assigning them a lower per-unit cost within the range of possible costs for that specific EGU type. We use several performance metrics to evaluate the magnitude of bias/error due to the overall cost model to ensure that the model reflects realistic dispatching scenarios. SO_x and NO_x emission factors [g/MWh] specific to each plant are obtained from eGRID2019.[10] Specific plant emission factors for primary $PM_{2.5}$, VOCs, and NH_3 could not be obtained from eGRID2019. Instead, power plant emission factors disaggregated by plant fuel type and prime mover technology, when possible, were sourced.[13,14] Due to the complexities of modeling stochastic wind and solar generation[15,16], only non-renewable readily dispatchable EGUs are assumed to be controlled within the OPF. Renewable plants are assumed to have fixed hourly generation based on their historic output.[10,11] Power plant ramping and unit commitment constraints are not accounted for in this version of the model but are recommended for future work.

3. *Exposure Reduction Strategies Analyzed*

Details regarding the development and assumptions of the different strategies are provided below.

Post Combustion NO_x and SO_x Emissions Control

In this strategy, coal, NG, and oil-powered power plants are retrofitted with high efficiency NO_x and SO_x removal technologies. Three different versions of this strategy are run: (1) only adopting SO_x removal technology in coal-fired power plants, (2) only adopting NOx removal technology is adopted in all fossil fuel-fired power plants, and (3) adopting both pollutant removal technologies are adopted for all fossil fuel-fired power plants. The EPA's National Electric Energy Data System (NEEDS) database[17] contains information regarding the type and removal efficiency of pollutant control technologies (if any) are installed in most EGUs. Data regarding power plant post combustion emission technologies are obtained from the EPA's Integrated Planning Model (IPM).[18] The IPM documentation shows that Limestone Forced Oxidation (LSFO) is the Flue Gas Desulfurization (FGD) technology with the highest removal efficiency of SO_x emitted from coal-fired power plants (up to 98% with a floor of 0.06 lbs/MMBtu). Using the NEEDS database, coal-fired power plants that do not have this type of scrubber installed are identified. Next, the SO_x emission rates of the identified power plants are adjusted to match the level they would be at if the LSFO scrubber were installed. For NO_x emissions, the IPM documentation shows that selective Catalytic Reduction (SCR) has the highest removal efficiency amongst all NO_x removal technologies: up to 90% removal with a floor of 0.06 b/MMBtu for coal-fired power plants and up to 80% removal for NG and oil-fired power plants. Like the SO_x based scenario, all power plants that do not have this type of technology installed are identified and have their NO_x emissions adjusted. The IPM documentation also holds

information for the additional operation costs [$/MWh] due to the use of the installed technologies as well as their capital costs of installation in [$/kW] disaggregated by plant heat rate and capacity. Operation costs of these plants are augmented with the data from IPM prior to rerunning the OPF. The capital costs required for their installation are also calculated. The ability to retrofit each EGU with emission control technologies was assumed to be possible without further investigation into any necessary installation prerequisites.

Higher Renewable Generation

The next strategy models the impacts of an increase in the renewable generation within the network, specifically from wind and solar sources. The 2019 baseline renewable generation is around 7.5% (eGRID). Three different scenarios are adopted to analyze the effects of integrating more renewables within the network. The first two are based on Illinois' renewable portfolio standards which assume 25% and 50% renewable generation by 2026 and 2040, respectively.[19] A third hypothetical scenario assumes 75% renewable generation by 2050 to model the effects of an aggressive renewable adoption. The expected increase in electricity demand (around a 1% increase annually) by the given years is factored in to reflect the amount of fossil-fuel based generation that will still be taking place during those years.[20] This allows the results to reflect the actual exposure damages that would still take place within those years while still running the OPF based on current load demand, allowing this scenario to be comparable to the others. To make the renewable scenarios comparable to the others, it is assumed that the expected increase in renewable generation capacity is adopted by the current grid conditions, so that all other factors (e.g., population numbers, underlying health risks, electricity demand, grid infrastructure) remains the same. Thus, we are not modeling future scenarios, rather what exposure would take place if future renewable generation capacity was present today. The expected renewable share

split between wind and solar is also accounted for based on EIA projections.[20] The increase in renewable generation within the OPF is modeled by increasing generation and capacity from existing solar and wind plants. Transmission equipment capacity is adjusted to handle the expected increased load from these sources. The capital costs of installation of the plants are quantified following a methodology outlined in Sergi et al.[21,22] The total required annual generation from these plants is quantified followed by the required installed capacity to meet generation demand using the plants' capacity factors. Capacity factors for wind and solar sources are derived the National Renewable Laboratory's (NREL) Wind Integration National Dataset (WIND)[23] and Global Horizontal Irradiance (GHI)[24] databases, respectively. To control for the rising intermittency issues from increased renewable generation capacity, installed utility-scaled battery storage capacity is also modeled following Sergi et al.[21,22] Additional battery storage leads to an increase in both operation and capital costs.

Relocation of High Polluting Plants to Low iF Zones

The final strategy models relocating high polluting EGUs to lower intake fraction (*iF*) census tracts. The results from the baseline run are used to rank order the EGUs based on their exposure contribution, and EGUs that lie within the 90th percentile are then identified. Illinois census tracts are then also rank ordered based on their *iF* values of each of the five $PM_{2.5}$ species. The intersection of census tracts that lie within the bottom 10th percentile of *iF* values of all five $PM_{2.5}$ species are chosen as areas to relocate the EGUs to. A new network is built with the assumption that these EGUs are relocated to those low *iF* census tracts. Next, three different versions of this scenario are run: the first assumes that the new EGUs have the same fuel type, operation costs, and emission rates of the ones they have replaced. The second and third versions assume that the replacement EGUs are either low-emitting EGUs of the same plant type and low-

emitting NG-fired power plants, respectively. Transmission equipment is added as needed to connect the new EGUs to the existing network. The capital costs of installing the EGUs[22] and transmission equipment[25] are also quantified. Assessing whether or not the new locations can actually host these EGUs from a regulatory perspective is beyond the scope of this study.

4. Linear Exposure Damages Factor

Given that nonlinear concentration-response functions are computationally difficult to integrate into the OPF model, linearized $PM_{2.5}$ intake effect and severity factor parameters[26] are used to approximate the exposure damages. The parameter used within the model is represented by ($\boldsymbol{\nu}$) which has units of [$ per kg$PM_{2.5}$ inhaled]. The exposure damages factor transforms $PM_{2.5}$ intake into premature deaths, and then monetizes the damages using the EPA recommended value of statistical life (VSL).[27] The development of this exposure damages factor is based on Fantke et al[26], who develop a set of global risk factors for policy assessments based on various $PM_{2.5}$ exposure-response models. The first parameter obtained from Fantke et al[26] used to develop exposure damages factor was a regionally specific effect factor which measures exposure health impacts in the form of disability-adjusted life years (DALY) per kg of $PM_{2.5}$ inhaled. The effect factor is combined with a mortality and morbidity severity factor, which is represented as a ratio of DALY per death for a specific region. The marginal effect factor and average severity factor obtained for the study's exposure domain are 228.9 DALY/kg$PM_{2.5}$ inhaled and 26.3 DALY/death, respectively. The VSL value assumed is $10M. Upon combining all these values together, the approximate exposure damages factor is found to be $\boldsymbol{\nu} \approx$ $80M per kg$PM_{2.5}$ inhaled. This factor is used to transform the induced per unit intake of each EGU [total kg$PM_{2.5}$

inhaled per MWh produced] into per unit monetized damages that can be used in the OPF [$ per MWh].

5. Exposure Domain

Figure S1 shows a map of the exposure domain and network EGUs.[10]

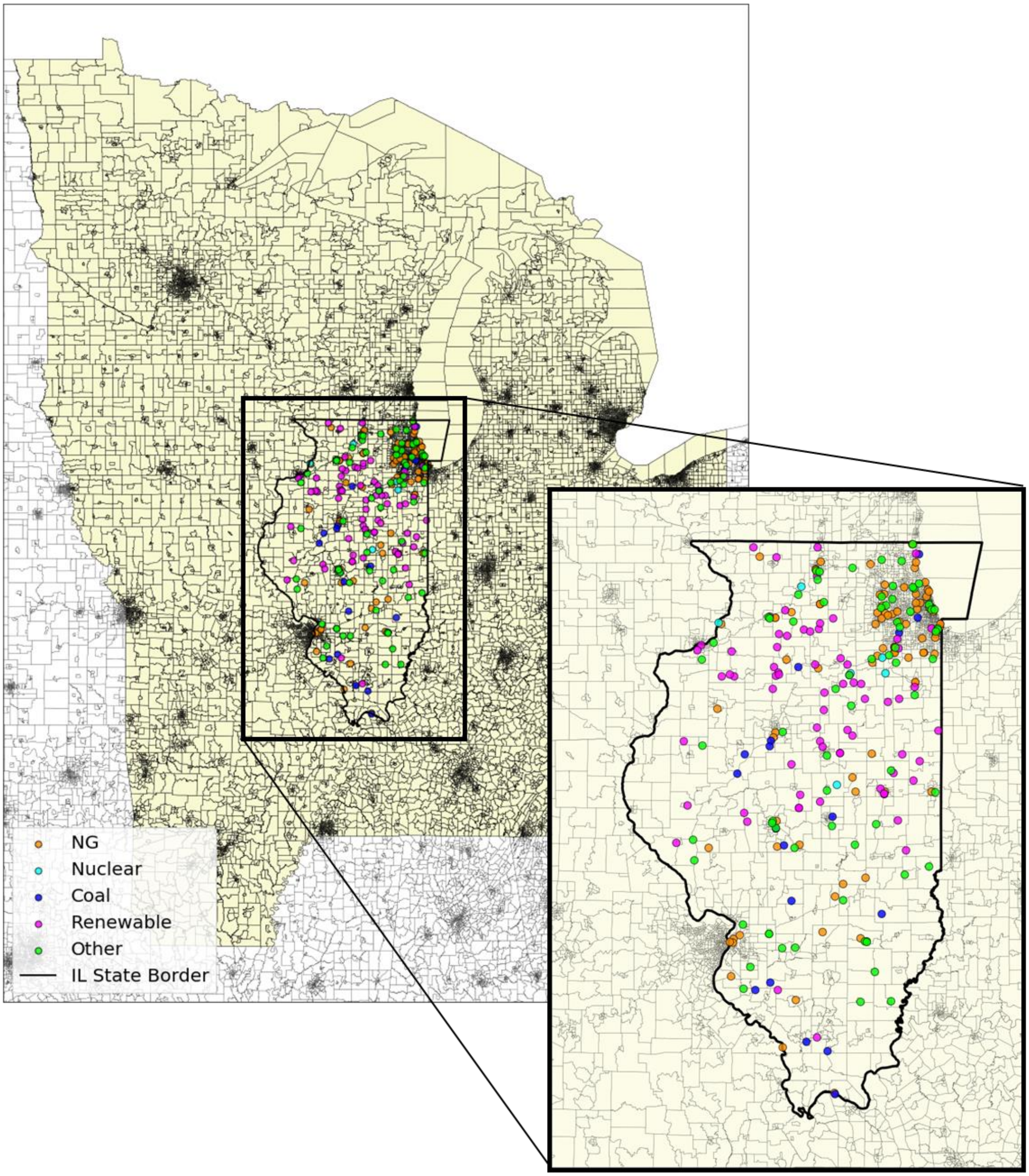

Figure S1: Exposure domain considered in the exposure-base optimal power flow (OPF) model in this study. Exposure is measured at the census tract level. The domain includes the state of

Illinois and its 10 neighboring states to capture the impacts of secondary $PM_{2.5}$ exposure: Arkansas, Indiana, Iowa, Kentucky, Michigan, Minnesota, Missouri, Ohio, Tennessee, and Wisconsin. Illinois based electricity generation units (EGUs) disaggregated by plant type (which represent the emissions sources in this study) are also highlighted.

6. Electricity Generation and Emission Metrics by EGU Plant Type

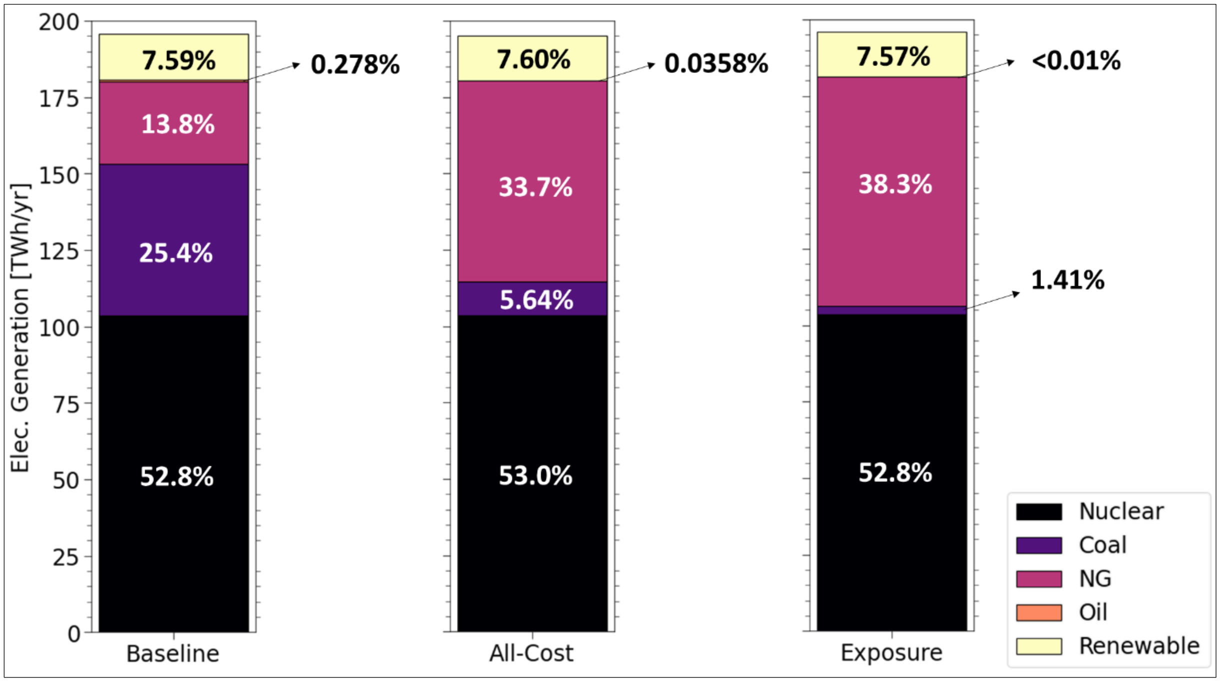

Figure S2: Annual electric generation [TWh/y] disaggregated by EGU plant type.

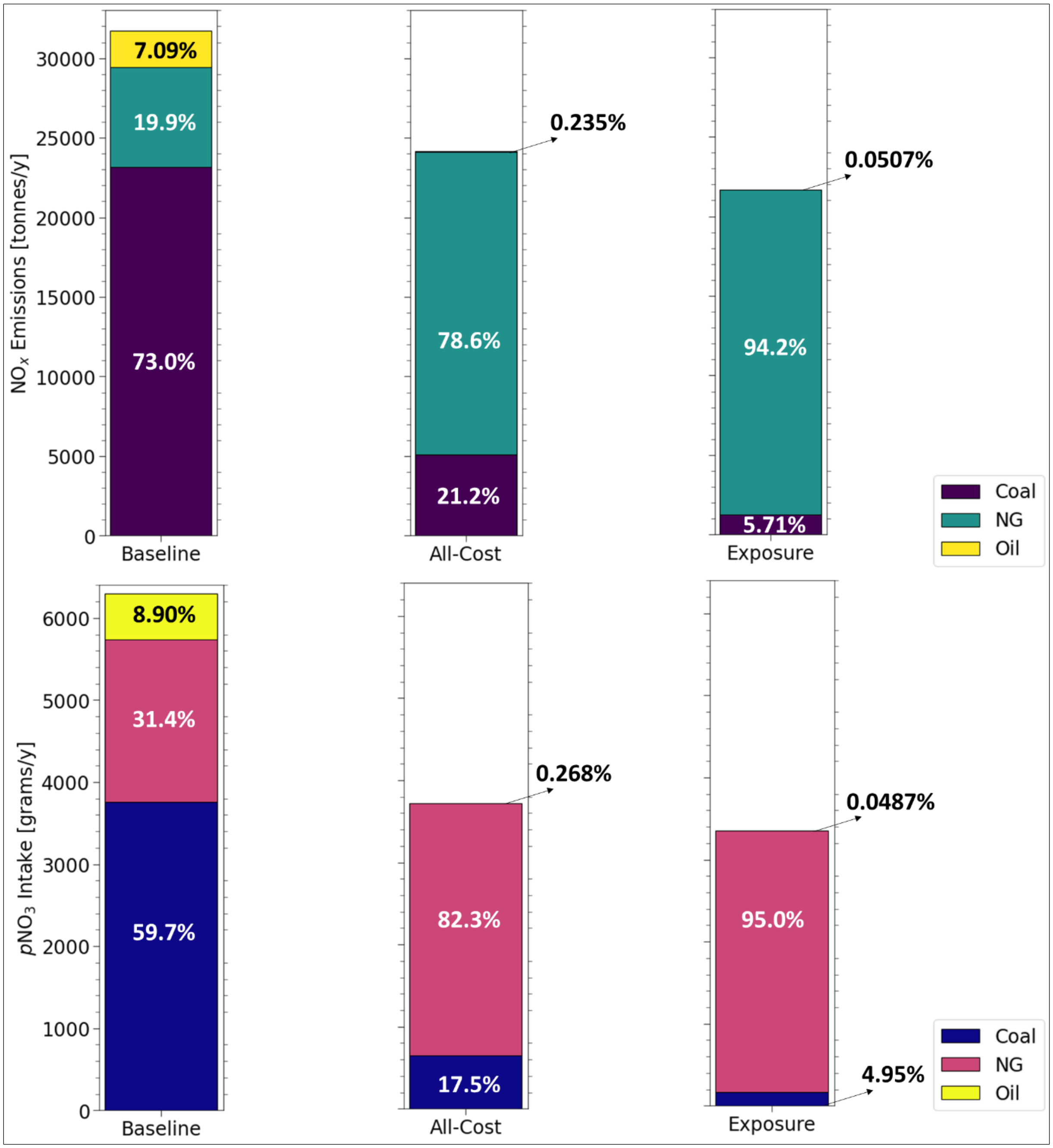

Figure S3: Annual NO_x emissions [tonnes/y] and pNO_3 intake [grams/y] disaggregated by EGU plant type.

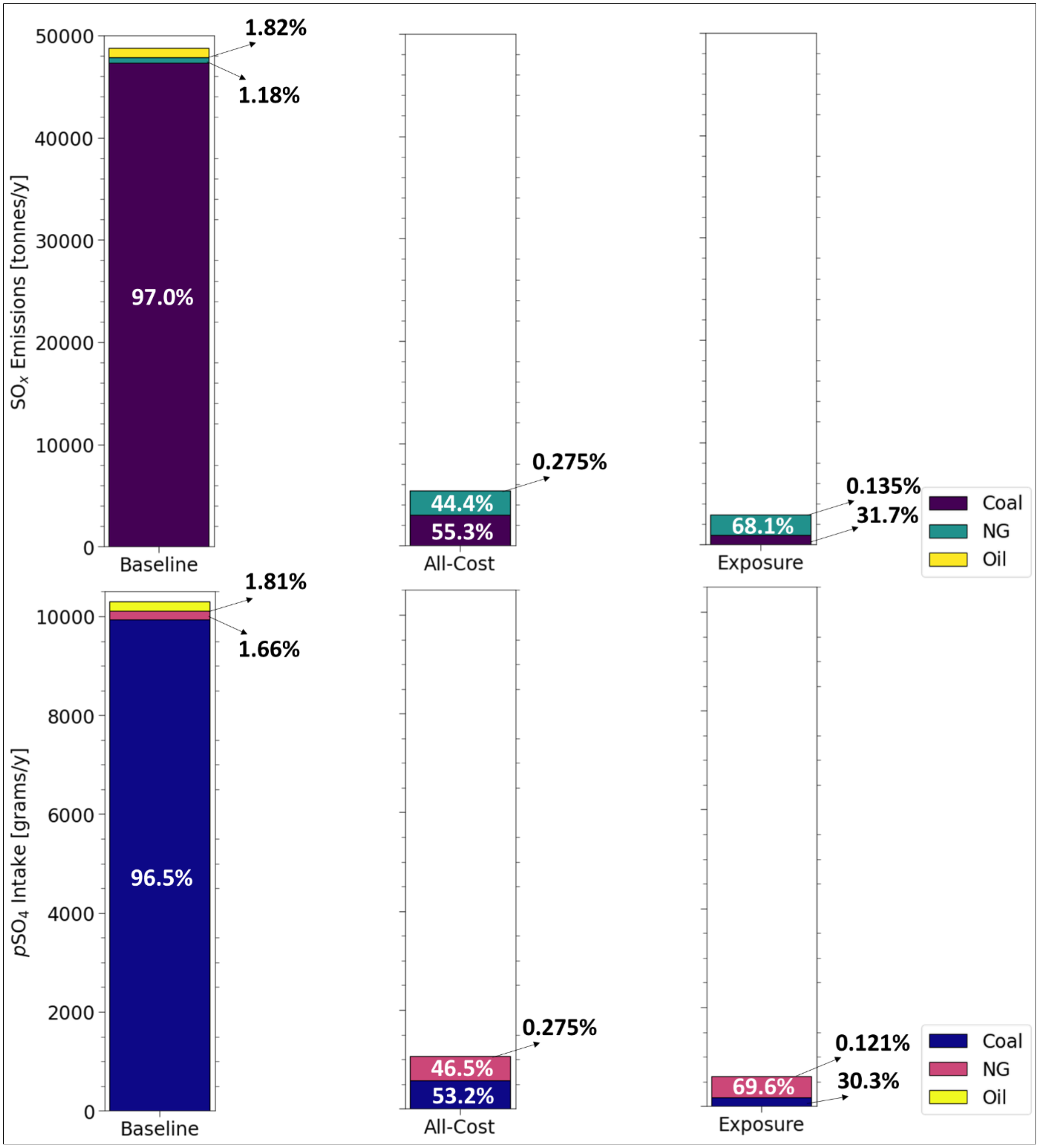

Figure S4: Annual SO_x emissions [tonnes/y] and $p$$SO_4$ intake [grams/y] disaggregated by EGU plant type.

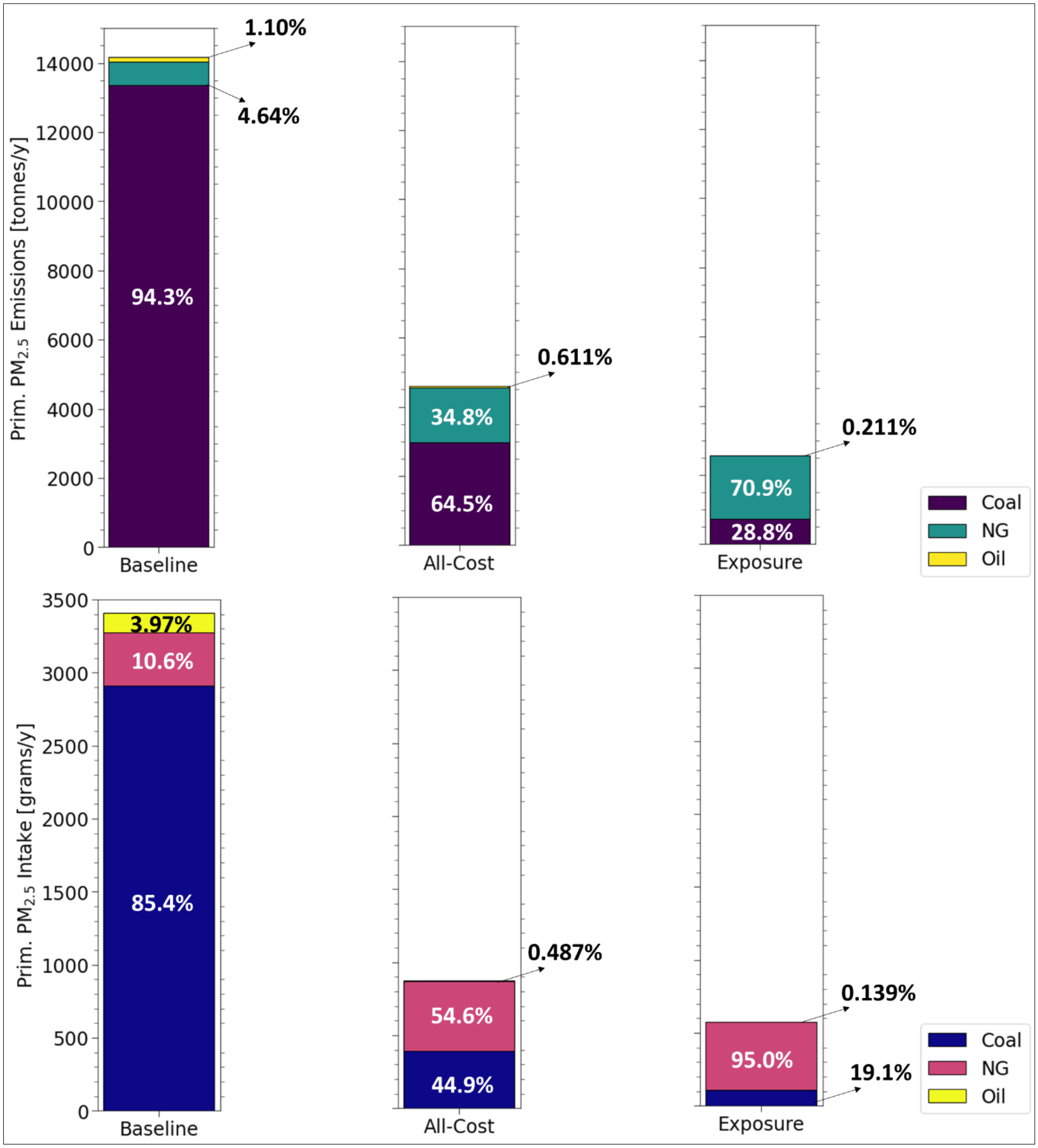

Figure S5: Primary $PM_{2.5}$ emissions [tonnes/y] and intake [grams/y] disaggregated by EGU plant type.

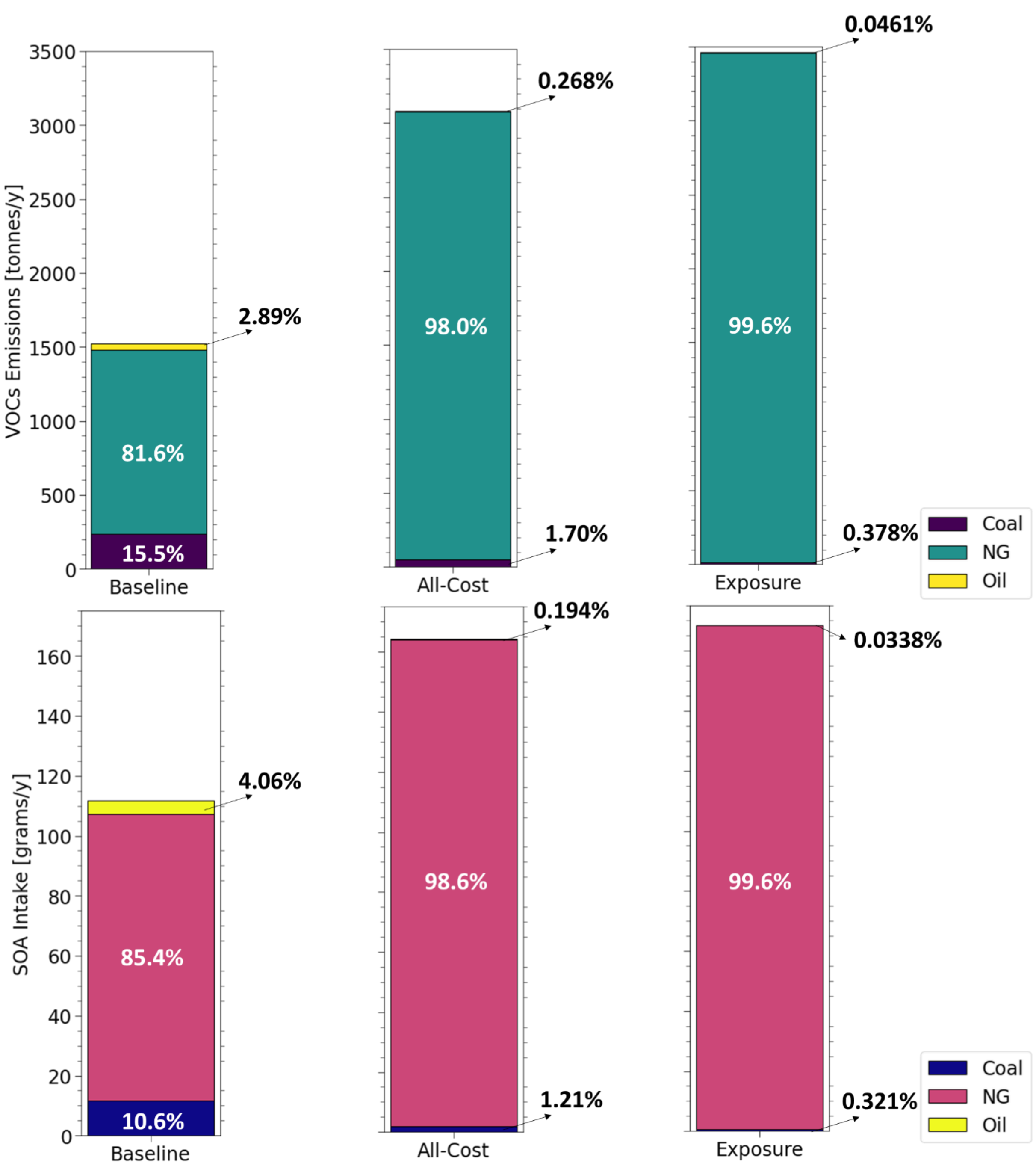

Figure S6: VOCs emissions [tonnes/y] and SOA intake [grams/y] disaggregated by EGU plant type.

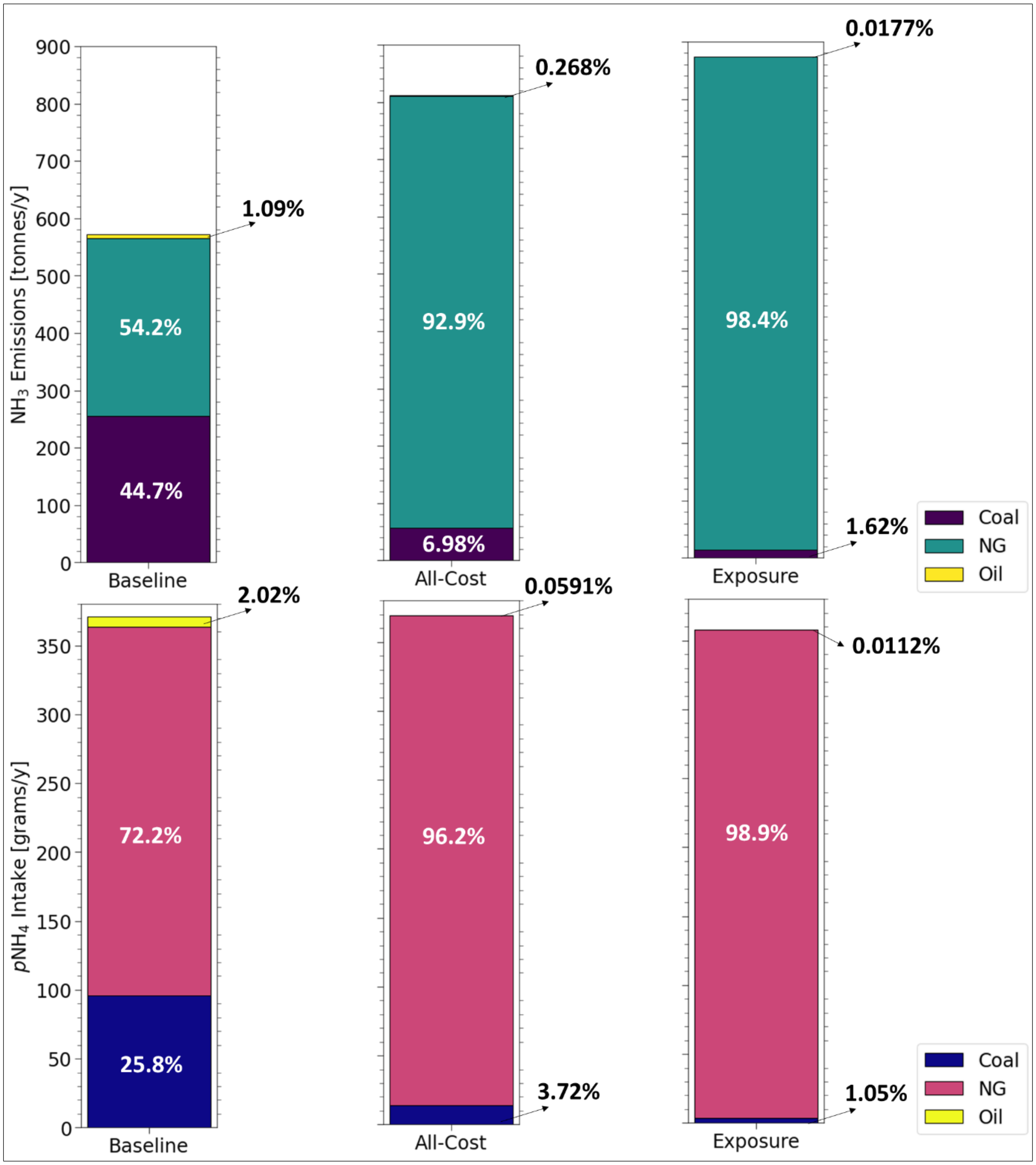

Figure S7: NH_3 emissions [tonnes/y] and $p$$NH_4$ intake [grams/y] disaggregated by EGU plant type.

7. Total Annualized Costs

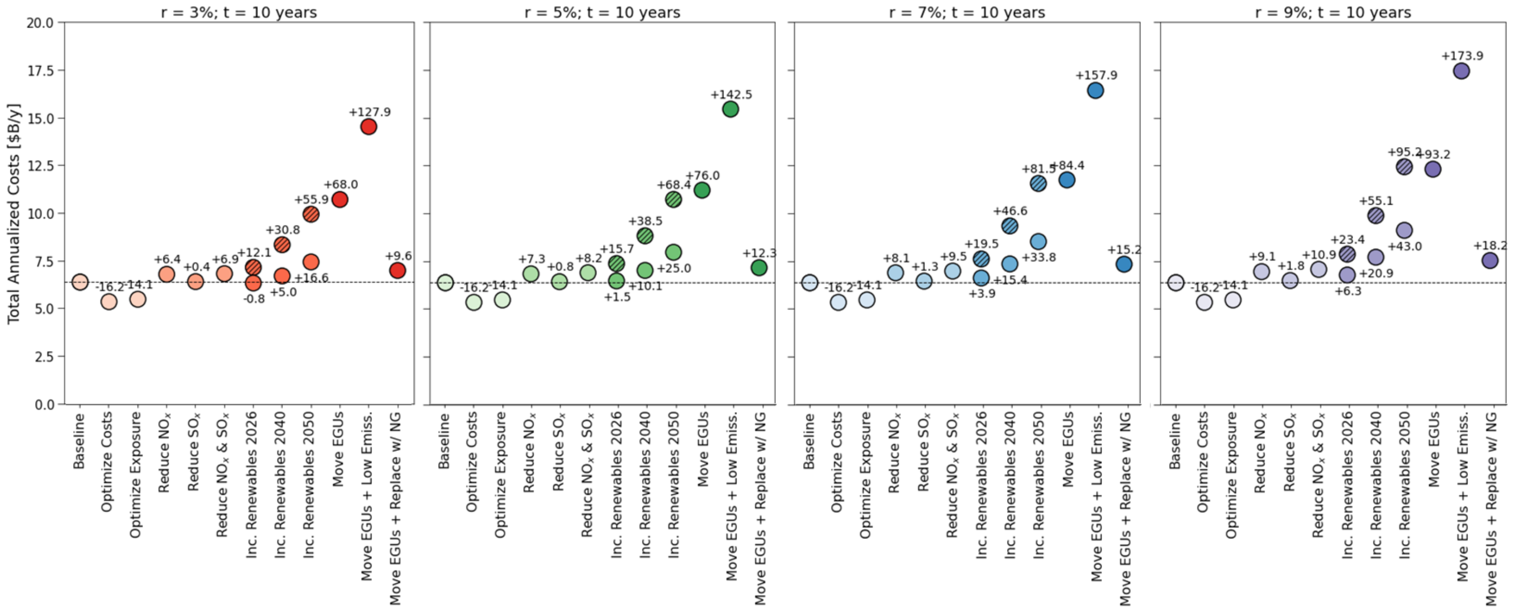

Figure S7: Total annualized costs of different scenarios and strategies assessed. Annualized costs reflect the sum of the sum equivalent annual cost of capital investments, operation dispatch costs, and exposure damages. (Discount rates: 3 – 9%; Average expected capital investment lifetime: 10 years).

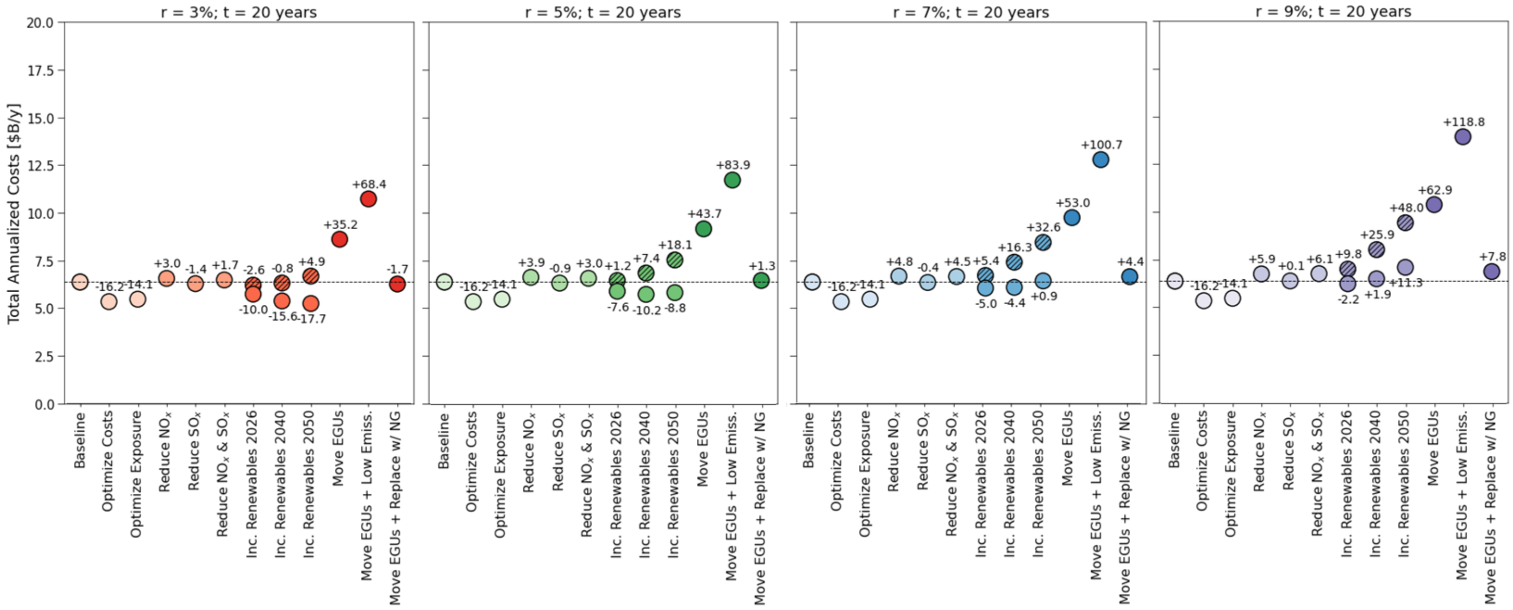

Figure S8: Total annualized costs of different scenarios and strategies assessed. Annualized costs reflect the sum of the sum equivalent annual cost of capital investments, operation dispatch costs, and exposure damages. (Discount rates: 3 – 9%; Average expected capital investment lifetime: 20 years).

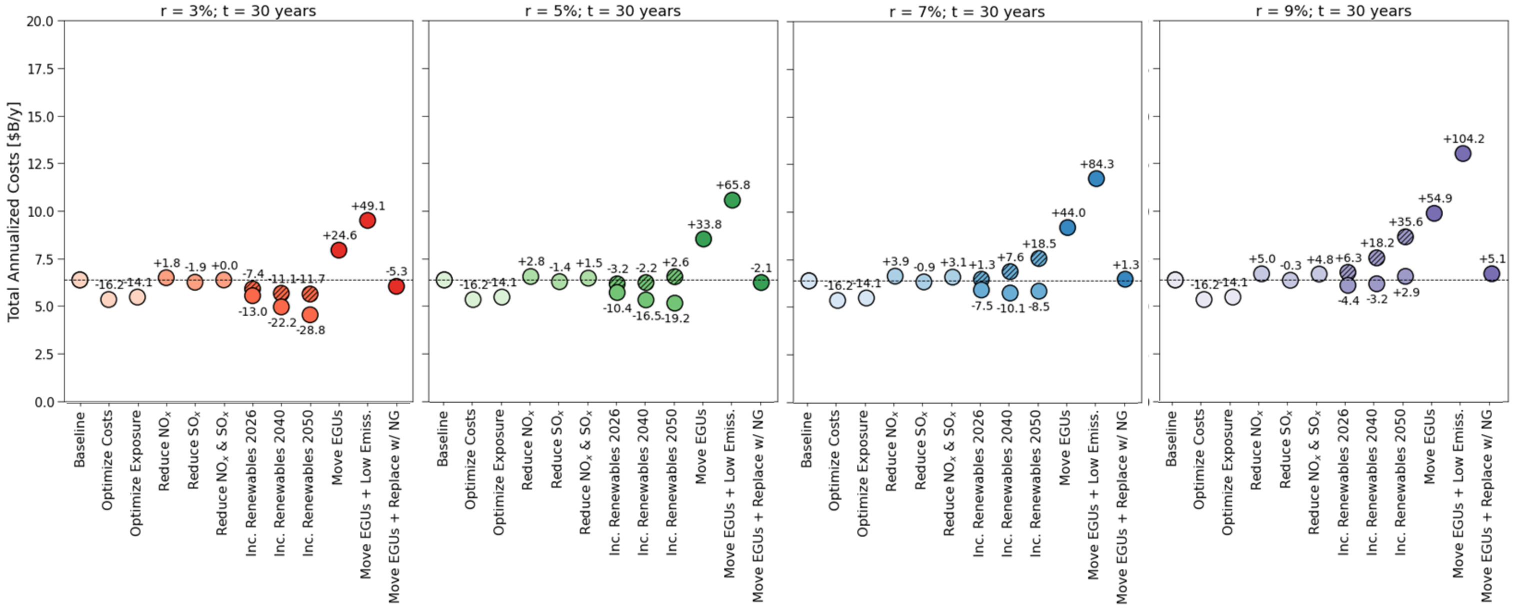

Figure S9: Total annualized costs of different scenarios and strategies assessed. Annualized costs reflect the sum of the sum equivalent annual cost of capital investments, operation dispatch costs, and exposure damages. (Discount rates: 3 – 9%; Average expected capital investment lifetime: 30 years).

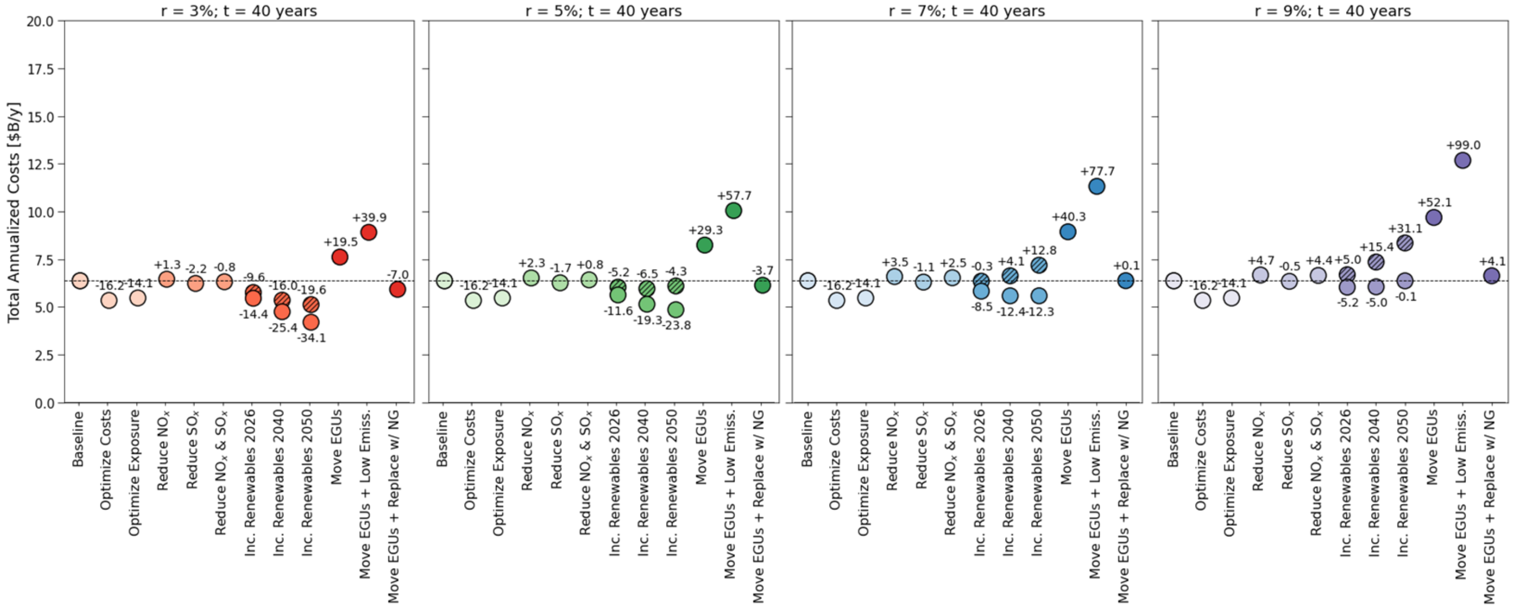

Figure S10: Total annualized costs of different scenarios and strategies assessed. Annualized costs reflect the sum of the sum equivalent annual cost of capital investments, operation dispatch costs, and exposure damages. (Discount rates: 3 – 9%; Average expected capital investment lifetime: 40 years).

8. Data Tables

Table S2: Performance metrics comparing monthly modeled electricity generation values against observed electricity generation values for the year 2019.

Metrics	JAN	FEB	MAR	APR	MAY	JUN	JUL	AUG	SEP	OCT	NOV	DEC	Total
Mean Observed Generation [GWh]	115	98	105	94	96	100	125	114	104	99	101	99	1000
Mean Simulated Generation [GWh]	95	95	78	82	77	87	100	94	90	79	88	79	1050
Standard deviation of Observed Generation [GWh]	327	290	317	304	308	306	323	320	301	311	312	319	3360
Standard deviation of Simulated Generation [GWh]	306	302	264	278	277	291	293	293	294	281	298	285	3440
Coefficient of determination (r^2)	0.985	0.989	0.987	0.986	0.988	0.982	0.980	0.978	0.989	0.987	0.984	0.986	0.995
Mean Bias [GWh]	1.62	12.1	-6.68	3.20	-1.79	2.29	-5.92	-1.96	3.53	-1.04	4.91	-1.33	41.9
Mean Fractional Bias [%]	-9.95	5.61	-8.47	18.6	-9.91	-17.2	11.5	-25.4	-20.5	-27.9	-6.26	-7.60	54.2
Normalized Mean Bias [%]	1.76	15.37	-7.93	4.23	-2.32	2.85	-5.90	-2.14	4.24	-1.31	6.06	-1.68	4.17
Root mean square error (RMSE) [GWh]	29.3	42.4	32.0	26.9	24.0	40.0	37.1	43.8	28.9	25.0	37.3	31.3	358
Mean Error [GWh]	7.18	12.4	7.01	6.71	4.25	10.2	10.3	9.48	8.78	5.00	9.44	4.04	105
Mean Fractional Error [%]	77.5	81.6	83.4	71.7	74.6	69.1	64.6	67.7	69.4	69.4	77.1	83.8	98.1
Normalized Mean Error [%]	7.79	15.7	8.32	8.86	5.48	12.7	10.3	10.4	10.5	6.29	11.7	5.10	10.5

Table S3: AC OPF exposure and cost results of the baseline run and the four $PM_{2.5}$ exposure mitigation strategies. Values in parentheses show % differences relative to the baseline run.

Description	Population Annual Intake [kg$PM_{2.5}$/y]	Population Weighted Concentration [μg/m^3]	Operation Costs [$B/y]	Exposure Damages [$B/y]	Total Costs [$B/y]
Baseline AC OPF	20.5	0.0492	4.51	1.75 – 1.97	6.26 – 6.49
Optimize (Minimize) Operation Costs + Exposure Damages	6.14 (-70.0%)	0.0148 (-70.0%)	4.79 (+6.08%)	0.526 – 0.583 (-70.1%)	5.31 – 5.37 (-16.2%)
Optimize (Minimize) Exposure Damages	4.65 (-77.3%)	0.0112 (-77.3%)	5.06 (+12.0%)	0.394 – 0.435 (-77.7%)	5.45 – 5.49 (-14.2%)
Reduce Post Combustion NO_x Emissions	18.8 (-8.15%)	0.0452 (-8.15%)	4.55 (+0.768%)	1.61 – 1.82 (-7.68%)	6.16 – 6.37 (-1.70%)
Reduce Post Combustion SO_x Emissions	17.2 (-15.9%)	0.0414 (-15.9%)	4.60 (+1.85%)	1.44 – 1.63 (-17.3%)	6.04 – 6.23 (-3.74%)
Reduce Post Combustion NO_x & SO_x Emissions	15.6 (-23.8%)	0.0375 (-23.6%)	4.63 (+2.50%)	1.32 – 1.49 (-24.3%)	5.95 – 6.12 (-5.35%)
Increase Renewable Generation (2026)	11.9 (-42.0%)	0.0285 (-42.0%)	3.89 (-13.8%)	0.998 – 1.12 (-43.1%)	4.89 – 5.00 (-22.4%)

Increase Renewable Generation (2026 w/ Battery Storage)	11.9 (-42.0%)	0.0285 (-42.0%)	3.94 (-12.6%)	0.998 – 1.12 (-43.1%)	4.94 - 5.06 (-21.5%)
Increase Renewable Generation (2040)	6.16 (-69.9%)	0.0148 (-69.9%)	3.09 (-31.6%)	0.501 – 0.558 (-71.5%)	3.58 – 3.64 (-43.3%)
Increase Renewable Generation (2040 w/ Battery Storage)	6.16 (-69.9%)	0.0148 (-69.9%)	3.21 (-28.9%)	0.501 – 0.558 (-71.5%)	3.71 – 3.76 (-41.4%)
Increase Renewable Generation (2050)	4.08 (-80.1%)	0.0098 (-80.1%)	1.98 (-56.1%)	0.313 – 0.350 (-82.1%)	2.29 – 2.33 (-63.7%)
Increase Renewable Generation (2050 w/ Battery Storage)	4.08 (-80.1%)	0.0098 (-80.1%)	2.21 (-51.1%)	0.313 – 0.350 (-82.1%)	2.52 – 2.55 (-60.2%)
Relocate High Polluting EGUs to Low iF Zones	11.7 (-42.6%)	0.0282 (-42.6%)	4.73 (+4.71%)	1.03 – 1.14 (-41.7%)	5.75 – 5.87 (-8.82%)
Relocate High Polluting EGUs to Low iF Zones + Replace with Adv. Low Emission EGUs	9.53 (-53.5%)	0.0229 (-53.5%)	4.78 (+5.87%)	0.806 – 0.896 (-54.2%)	5.59 – 5.67 (-11.6%)
Relocate High Polluting EGUs to Low iF Zones + Replace with Adv. Low Emission NG	7.87 (-61.6%)	0.0189 (-61.6%)	4.64 (+2.77%)	0.634 – 0.701 (-64.1%)	5.27 – 5.34 (-16.7%)

Table S4: Comparing simulation results from the DC optimal power flow approximation to the AC optimal power flow model run. Results are shown for the DC run and the absolute and relative increase in values is displayed for the AC model run.

	DC Optimal Power Flow	ΔAC Optimal Power Flow
Annual Generation (TWh)	174	+7.00 (+4.03%)
Illinois Population Weighted Concentration (μg/m^3)	0.152	+0.00751 (+4.93%)
All Population Weighted Concentration (μg/m^3)	0.0475	+0.00170 (+3.59%)
Illinois Population Annual Intake (kgPM$_{2.5}$/y)	10.6	+0.523 (+4.93%)
All Population Annual Intake (kgPM$_{2.5}$/y)	19.8	+0.709 (+3.59%)
Annual Deaths	180 – 203	+6.00 – +7.00 (+3.35%)
Exposure Damages ($B/y)	1.69 – 1.91	+0.0565 – +0.0640 (+3.35%)
Operation Costs ($B/y)	4.42	+0.164 (+3.72%)
Total Costs ($B/y)	6.11 – 6.32	+0.221 – +0.228 (+3.62%)

8. References

(1) Frank, S.; Rebennack, S. An Introduction to Optimal Power Flow: Theory, Formulation, and Examples. *IIE Trans.* **2016**, *48* (12), 1172–1197. https://doi.org/10.1080/0740817X.2016.1189626.

(2) Gegner, K. M.; Birchfield, A. B.; Xu, T.; Shetye, K. S.; Overbye, T. J. A Methodology for the Creation of Geographically Realistic Synthetic Power Flow Models. In *2016 IEEE Power and Energy Conference at Illinois (PECI)*; 2016; pp 1–6. https://doi.org/10.1109/PECI.2016.7459256.

(3) Birchfield, A. B.; Xu, T.; Gegner, K. M.; Shetye, K. S.; Overbye, T. J. Grid Structural Characteristics as Validation Criteria for Synthetic Networks. *IEEE Trans. Power Syst.* **2017**, *32* (4), 3258–3265. https://doi.org/10.1109/TPWRS.2016.2616385.

(4) Birchfield, A. B.; Xu, T.; Overbye, T. J. Power Flow Convergence and Reactive Power Planning in the Creation of Large Synthetic Grids. *IEEE Trans. Power Syst.* **2018**, *33* (6), 6667–6674. https://doi.org/10.1109/TPWRS.2018.2813525.

(5) Thurner, L.; Scheidler, A.; Schäfer, F.; Menke, J.-H.; Dollichon, J.; Meier, F.; Meinecke, S.; Braun, M. Pandapower—An Open-Source Python Tool for Convenient Modeling, Analysis, and Optimization of Electric Power Systems. *IEEE Trans. Power Syst.* **2018**, *33* (6), 6510–6521. https://doi.org/10.1109/TPWRS.2018.2829021.

(6) Homeland Infrastructure Foundation-Level Data (HIFLD). Electric Power Transmission Lines. **2022**. https://hifld-geoplatform.opendata.arcgis.com/datasets/electric-power-transmission-lines-1/about. (Accessed on June 5, 2022).

(7) Homeland Infrastructure Foundation-Level Data (HIFLD). Electric Substations. **2022**. https://hifld-geoplatform.opendata.arcgis.com/datasets/electric-substations-1/about. (Accessed on June 5, 2022).

(8) Birchfield, A. B.; Xu, T.; Gegner, K. M.; Shetye, K. S.; Overbye, T. J. Grid Structural Characteristics as Validation Criteria for Synthetic Networks. *IEEE Trans. Power Syst.* **2017**, *32* (4), 3258–3265. https://doi.org/10.1109/TPWRS.2016.2616385.

(9) Homeland Infrastructure Foundation-Level Data (HIFLD). Electric Retail Service Territories. **2022**. https://hifld-geoplatform.opendata.arcgis.com/datasets/geoplatform::electric-retail-service-territories-1/about. (Accessed on June 5, 2022).

(10) U.S. Environmental Protection Agency. Emissions & Generation Integrated Database (EGRID) 2019 - Data. **2022**. https://www.epa.gov/egrid/download-data. (Accessed on June 5, 2022).

(11) U.S. Environmental Protection Agency. Continuous Emissions Monitoring Systems (CEMS) Index of /DmDnLoad/Emissions/Hourly/Monthly/2019. **2022**. https://gaftp.epa.gov/DmDnLoad/emissions/hourly/monthly/2019/.(Accessed on June 5, 2022).

(12) U.S. Energy Information Administration. Hourly Electric Grid Monitor: Midcontinent Independent System Operator, Inc. (MISO) Electricity Overview. **2022**. https://www.eia.gov/electricity/gridmonitor/dashboard/electric_overview/balancing_authority/MISO. (Accessed on June 5, 2022).

(13) Cai, H.; Wang, M.; Elgowainy, A.; Han, J. *Updated Greenhouse Gas and Criteria Air Pollutant Emission Factors and Their Probability Distribution Functions for Electricity*

Generating Units; ANL/ESD/12-2; Argonne National Lab. (ANL), Argonne, IL (United States), 2012. https://doi.org/10.2172/1045758.

(14) U.S. Environmental Protection Agency. Emission Factors and Inventory Group - Estimating Ammonia Emissions from Anthropogenic Nonagricultural Sources. **2004**. https://www.epa.gov/sites/production/files/2015-08/documents/eiip_areasourcesnh3.pdf. (Accessed on June 5, 2022).

(15) Biswas, P. P.; Suganthan, P. N.; Amaratunga, G. A. J. Optimal Power Flow Solutions Incorporating Stochastic Wind and Solar Power. *Energy Convers. Manag.* **2017**, *148*, 1194–1207. https://doi.org/10.1016/j.enconman.2017.06.071.

(16) Lubin, M.; Dvorkin, Y.; Backhaus, S. A Robust Approach to Chance Constrained Optimal Power Flow With Renewable Generation. *IEEE Trans. Power Syst.* **2016**, *31* (5), 3840–3849. https://doi.org/10.1109/TPWRS.2015.2499753.

(17) U.S. Environmental Protection Agency. National Electric Energy Data System (NEEDS) V6. **2022**. https://www.epa.gov/power-sector-modeling/national-electric-energy-data-system-needs-v6. (Accessed on June 5, 2022).

(18) U.S. Environmental Protection Agency. Documentation for Integrated Planning Model (IPM) Base Case v.4.10. **2022**. https://www.epa.gov/power-sector-modeling/documentation-integrated-planning-model-ipm-base-case-v410. (Accessed on June 5, 2022).

(19) U.S. Energy Information Administration. RPS (Renewable Portfolio Standards). **2022**. https://www.eia.gov/todayinenergy/index.php?tg=RPS%20(Renewable%20Portfolio%20Standards). (Accessed on June 5, 2022).

(20) U.S. Energy Information Administration. Annual Energy Outlook 2022. **2022**. https://www.eia.gov/outlooks/aeo/.(Accessed on June 5, 2022).

(21) Deetjen, T. A.; Azevedo, I. L. Climate and Health Benefits of Rapid Coal-to-Gas Fuel Switching in the U.S. Power Sector Offset Methane Leakage and Production Cost Increases. *Environ. Sci. Technol.* **2020**, *54* (18), 11494–11505. https://doi.org/10.1021/acs.est.9b06499.

(22) National Renewable Energy Laboratory. Annual Technology Baseline: 2022 Electricity ATB Technologies. **2022**. https://atb.nrel.gov/electricity/2022/technologies. (Accessed on June 5, 2022).

(23) National Renewable Energy Laboratory. Wind Integration National Dataset (WIND) Toolkit Power Data Site Index. **2022**. https://data.nrel.gov/submissions/54.

(24) National Renewable Energy Laboratory. Solar Resource Maps and Data. **2022**. https://www.nrel.gov/gis/solar-resource-maps.html. (Accessed on June 5, 2022).

(25) Midcontinent Independent System Operator. Transmission Cost Estimation Guide MTEP19. **2022**. https://cdn.misoenergy.org/20190212%20PSC%20Item%2005a%20Transmission%20Cost%20Estimation%20Guide%20for%20MTEP%202019_for%20review317692.pdf. (Accessed on June 5, 2022).

(26) Fantke, P.; McKone, T. E.; Tainio, M.; Jolliet, O.; Apte, J. S.; Stylianou, K. S.; Illner, N.; Marshall, J. D.; Choma, E. F.; Evans, J. S. Global Effect Factors for Exposure to Fine Particulate Matter. *Environ. Sci. Technol.* **2019**, *53* (12), 6855–6868. https://doi.org/10.1021/acs.est.9b01800.

(27) U.S. Environmental Protection Agency. Mortality Risk Valuation. **2022**. https://www.epa.gov/environmental-economics/mortality-risk-valuation. (Accessed on June 5, 2022).
